# Supplementary material for: One-step synthesis of single-site vanadium substitution in 1T-WS2 monolayers for enhanced hydrogen evolution catalysis
Source: Nat Commun. 2021 Jan 29;12:709. doi: 10.1038/s41467-021-20951-9 (PMC7846562; doi:10.1038/s41467-021-20951-9)
Supplement: Supplementary file 1 — Supplementary Information [file 41467_2021_20951_MOESM1_ESM.pdf]

*Supporting Information*

# One-Step Synthesis of Single-Site Vanadium Substitution in 1T-$WS_2$ Monolayers for Enhanced Hydrogen Evolution Catalysis

Ali Han[1,2#], Xiaofeng Zhou[1,3#], Xijun Wang[4#], Sheng Liu[5], Qihua Xiong[6], Qinghua Zhang[7], Lin Gu[7], Zechao Zhuang,[2] Wenjing Zhang[8], Fanxing Li[4], Dingsheng Wang[2*], Lain-Jong Li[1*], Yadong Li[2]

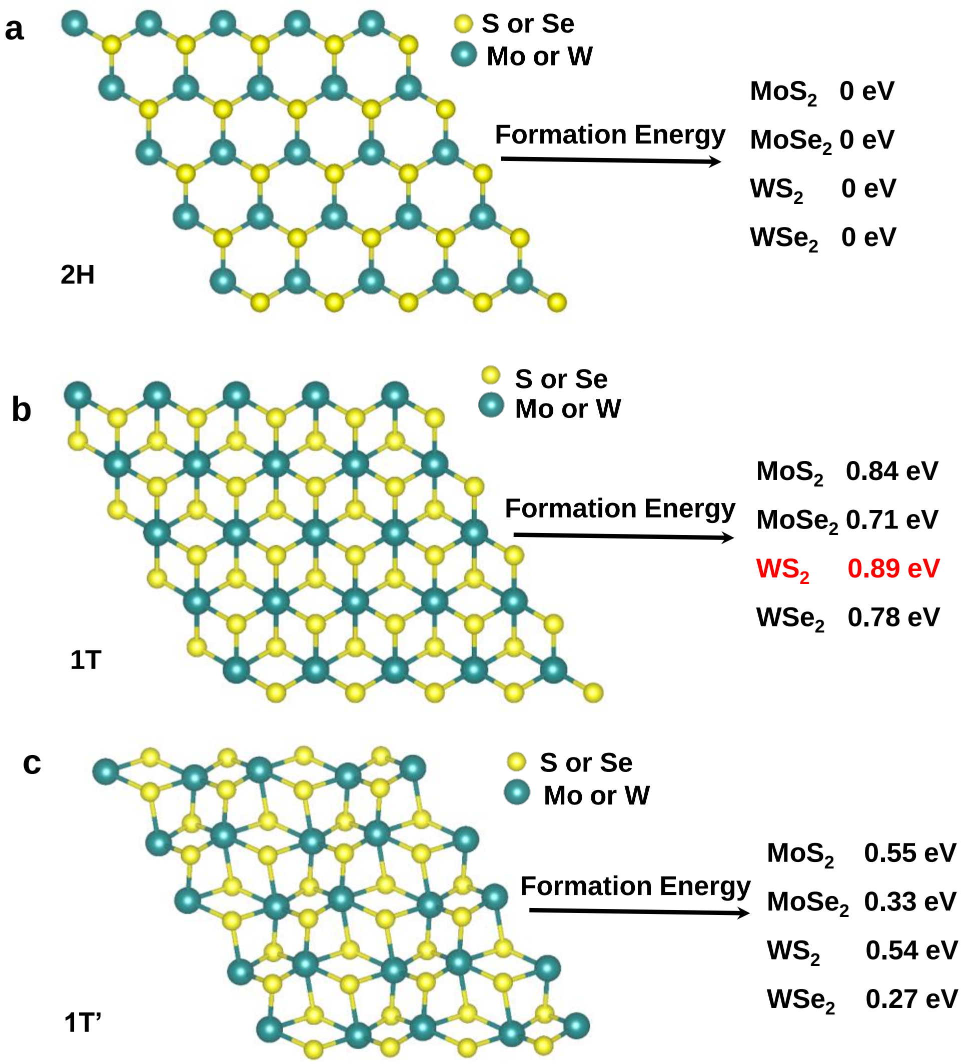


**Supplementary Fig. 1** Schematic models of single-layered $MX_2$ with 2H (a), 1T (b) and 1T' (c) phases in basal plane and the corresponding formation energies.[1]

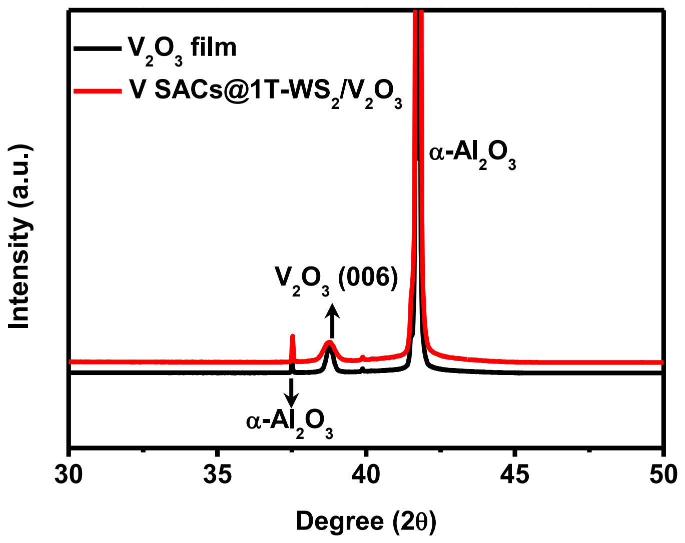


**Supplementary Fig. 2** XRD patterns of $V_2O_3$ film and V SACs@1T-$WS_2$/$V_2O_3$ film.

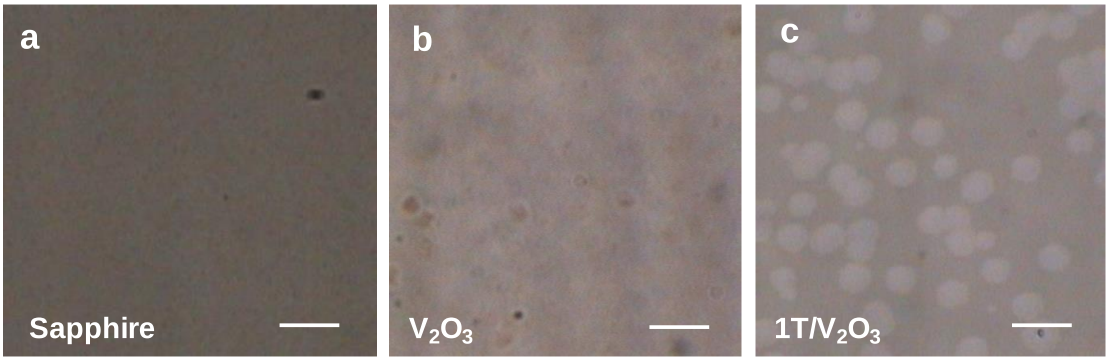


**Supplementary Fig. 3 a-c**, Optical micrographs of fresh sapphire (a), as-grown $V_2O_3$ film (b), 1T/$V_2O_3$ film (c) by CVD. Scale bars: a-c, 60 μm.

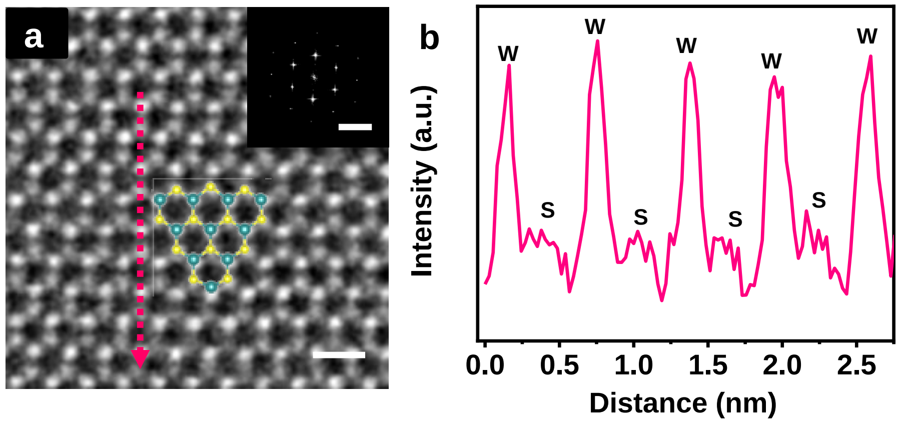


**Supplementary Fig. 4 a,** High-resolution STEM image of monolayer 2H-$WS_2$. The blue spheres and yellow spheres represent the W atoms and S atoms, respectively, in the schematic model of 2H-$WS_2$ in Figure S4a. Scale bar: 0.5 nm. Inset: the corresponding Fast Fourier Transform (FFT) of Figure S4a. Scale bar: 5 1/nm; **b**, Intensity sequence profile of W-S-W (pink dashed arrow indicated by Figure S4a).

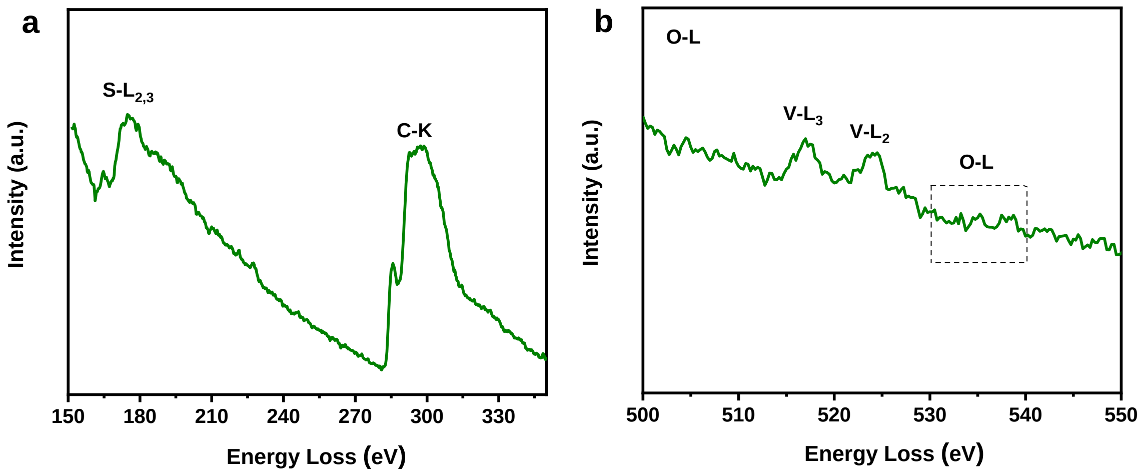


**Supplementary Fig. 5** EELS spectra of S (a) and O (b) in the V SACs@1T-$WS_2$ monolayer. The carbon detection was associated with remaining PMMA polymer during the transferring process.

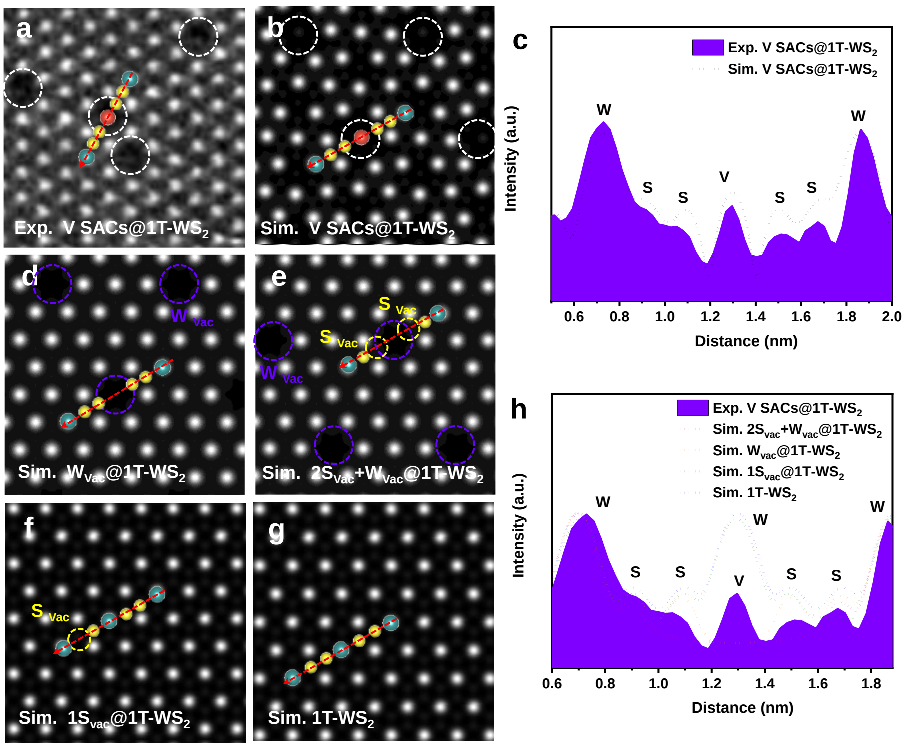


**Supplementary Fig. 6 a-b,** Experimental (a) and simulated (b) STEM images of V SACs@1TWS$_2$, respectively; **c,** Corresponding experimental (purple) and simulated (black dots) intensity sequence profiles of W-S-S-V-S-S-W (red dashed arrow) indicated by the STEM images. The white dashed circles represent the V atoms; **d-e,** Simulated STEM images of $W_{vac}$@1T-WS$_2$ with no S vacancy (d) and 2S vacancies (e). The purple dashed circles and yellow dashed circles represent the $W_{vac}$ and $S_{vac}$, respectively; **f-g,** Simulated STEM images of 1T-WS$_2$ with 1S vacancy (f) and no S vacancy (g); **h,** Corresponding experimental (purple) intensity sequence profile of W-S-S-V-S-S-W (red dashed arrow) indicated by Figure S6a, simulated (orange dots) intensity sequence profiles of W-S-S-$W_{vac}$-S-S-W (red dashed arrow) indicated by the Figure S6d, simulated (pink dots) intensity sequence profiles of W-S-$S_{vac}$-$W_{vac}$-$S_{vac}$-S-W (red dashed arrow) indicated by the Figure S6e, simulated (olive dots) intensity sequence profiles of W-S-S-W-S-$S_{vac}$-W (red dashed arrow) indicated by the Figure S6f and simulated (blue dots) intensity sequence profiles of W-S-S-W-S-S-W (red dashed arrow) indicated by the Figure S6g. The blue spheres, red spheres and yellow spheres represent the W atoms, V atoms and S atoms, respectively.

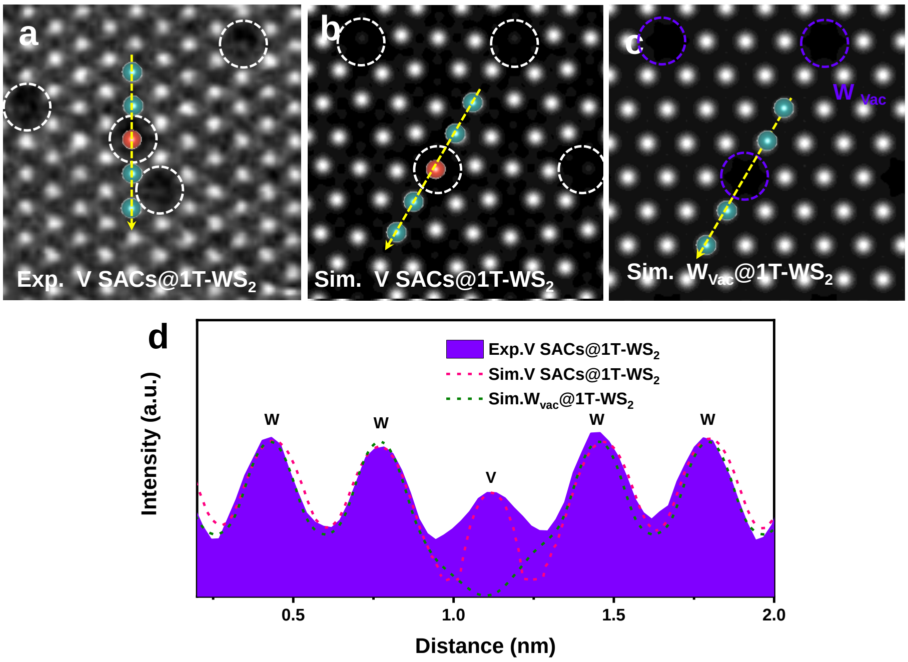


**Supplementary Fig. 7 a-b,** Experimental (a) and simulated (b) STEM images of V SACs@1T-$WS_2$, respectively. The white dashed circles represent the V atoms; **c,** Simulated STEM image of 1T-$WS_2$ with vacancies at W sites ($W_{vac}$@1T-$WS_2$). The purple dashed circles represent the vacancies at W sites; **d,** Corresponding intensity sequence profiles of W-W-V-W-W or W-W-$W_{vac}$-W-W indicated by the STEM images (yellow dashed arrow). The blue spheres, and red spheres in Figure S7a-7c represent the W atoms and V atoms, respectively.

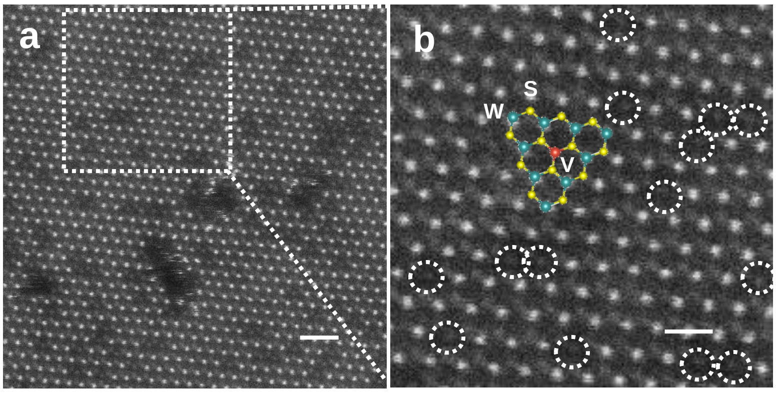


**Supplementary Fig. 8 a-b,** High-resolution HAADF-STEM images of monolayered V SACs@1T-$WS_2$ after annealing at 200 ºC in air for 30 mins. The blue spheres, red spheres and yellow spheres represent the W atoms, V atoms and S atoms, respectively, in the schematic model of V SACs@2H-$WS_2$ in Figure S8b. The V atoms are highlighted by the white dashed circles. Please note that the V SACs@1T-$WS_2$ on the $V_2O_3$ film cannot be directly annealed in air, as $V_2O_3$ film will be transformed into $VO_2$ (M) above 300°C (see Figure S9). Scale bars: a, 1 nm; b, 0.5 nm.

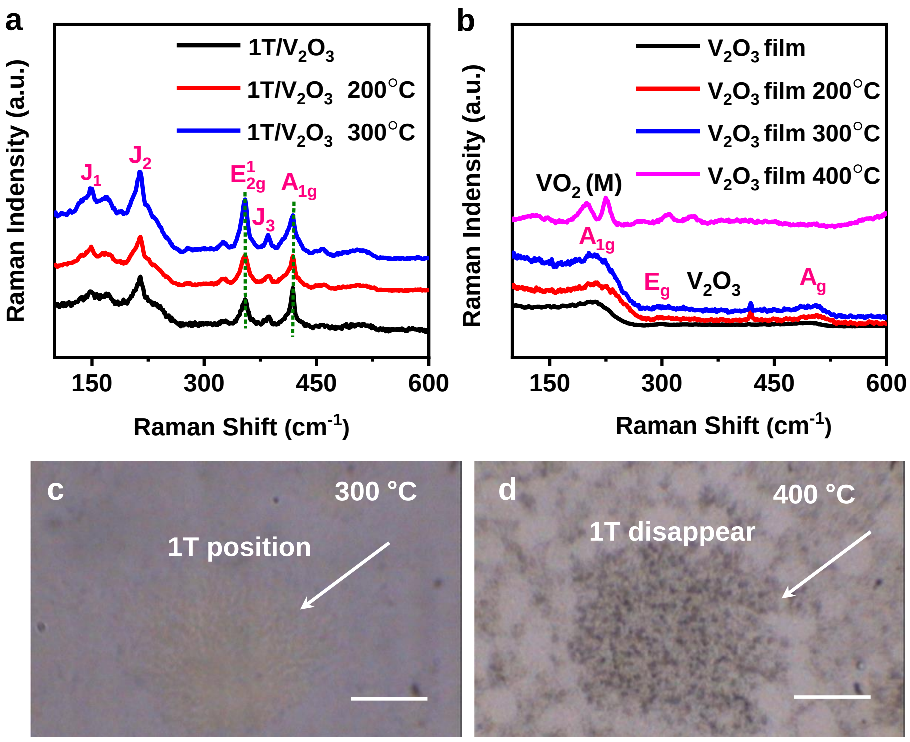


**Supplementary Fig. 9 a,** Raman spectra of 1T-$V_2O_3$ region with different annealing temperatures. 1T features in Raman spectra get relatively stable signals with the annealing temperature range from 25 ºC to 300 ºC in air; **b,** Raman spectra of $V_2O_3$ region with different annealing temperatures. The $V_2O_3$ film becomes $VO_2$ (M) at 400 ºC in air for 5 mins; **c-d,** Optical micrographs of as-grown 1T-$V_2O_3$ phase on $V_2O_3$ film after annealing under different temperatures for 5 mins. 300 ºC (c) and 400 ºC (d). Scale bars: c-d, 10 μm.

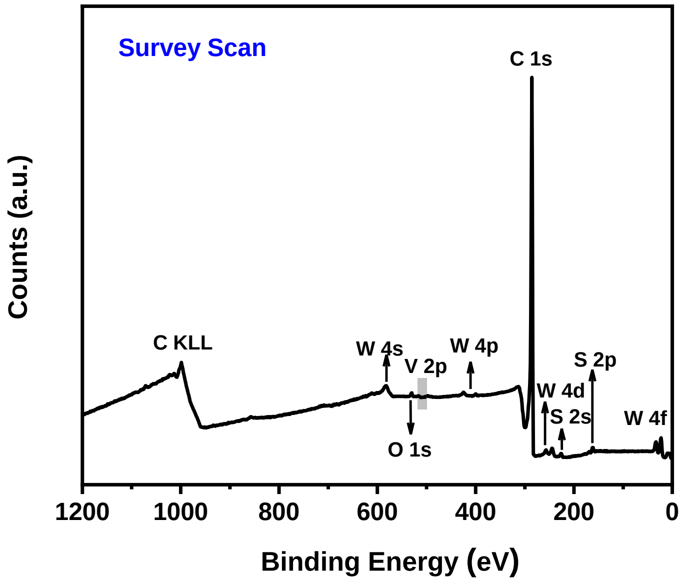


**Supplementary Fig. 10** Survey scan of V SACs@1T-$WS_2$ transferred on HOPG substrate.

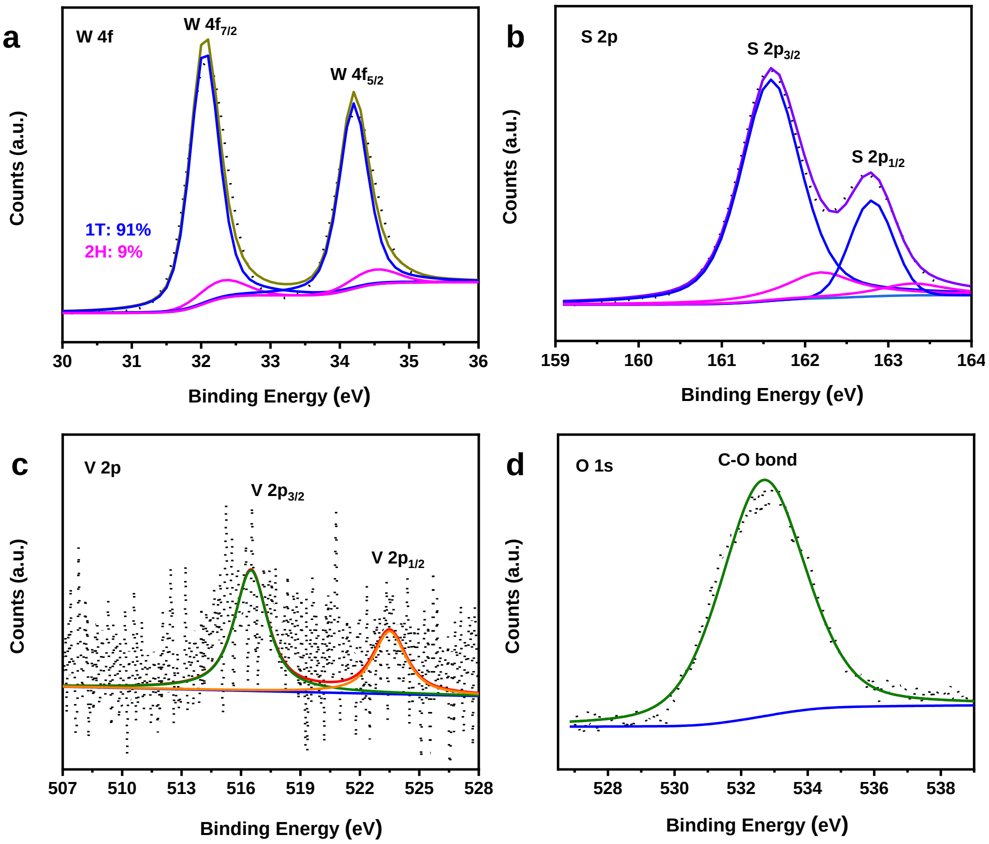


**Supplementary Fig. 11 a-d**, High-resolution XPS spectra of W 4f (a), S 2p (b), V 2p (c) and O 1s (d) core levels of V SACs@1T-$WS_2$ transferred on HOPG substrate. The fitting blue and pink curves represent the contributions of 1T and 2H phases in Fig. S11 a-b, respectively. The molar ratio of W/V obtained by the XPS analysis was ~95:5.

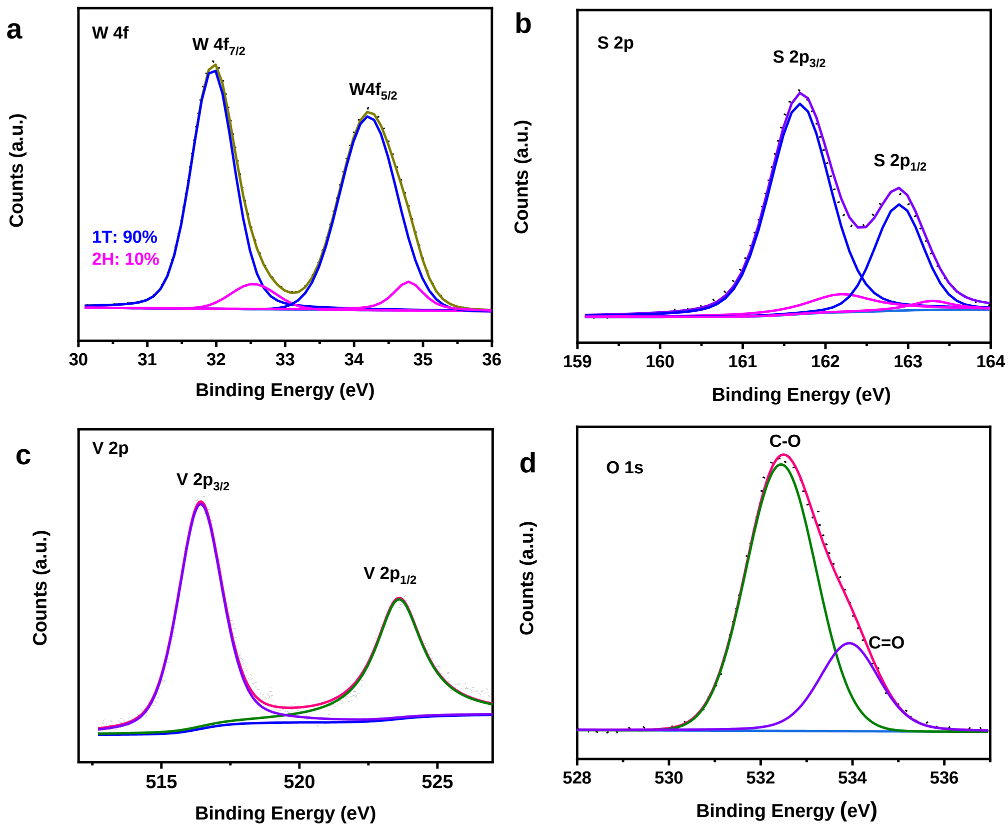


**Supplementary Fig. 12** a-d, High-resolution XPS spectra of W 4f (a), S 2p (b), V 2p (c) and O 1s (d) core levels of V SACs@1T-$WS_2$ transferred on HOPG substrate for 15 times. The fitting blue and pink curves represent the contributions of 1T and 2H phases in Fig. S12 a-b, respectively. The molar ratio of W/V obtained by the XPS analysis was ~16:1.

To exclude the possibility of $VS_2$ and $V_2O_3$/$VO_2$ contaminations in the transferred V SACs@1T-$WS_2$ monolayers, we transferred V SACs@1T-$WS_2$ monolayers on the HOPG substrate for the further XPS analyses. The survey scan and high-resolution XPS spectra of W 4f, S 2p, V 2p and O 1s were shown in Figure S10 and Figure S11, respectively. Except for the signals of W4f and S 2p, the V 2p signals were detected after a long-term acquisition time during the XPS scanning with much weaker intensity than W 4f (Fig. S11a) and S 2p (Fig. S11b), which should be caused by the low atomic density of V atoms in the 1T sample. After deconvoluting the V 2p signals, the peaks at 516.4 eV and 523.5 eV were assigned to $V^{4+}$ from V-S bond in 1T sample.[2-6] As the XPS technique was able to detect the surface of sample in 10 nm depth. To further verify the V signal in the 1T sample, we transferred the 1T samples on the same HOPG area for 15 times to acquire the XPS spectra. As shown in Figure S12c, an enhanced V 2p signal was observed, matching well with the V 2p signal in Figure S11c.

Please note that if V-based materials were present in the transferred sample, a prominent V 2p peak should be detected. However, only weak V signals were detected, indicating no V-based contamination found in the transferred samples. Importantly, if the V-based contaminations (e.g. $V_2O_3$, $VO_2$, $VS_2$) were contained in the transferred 1T sample, obviously enhanced V/W molar ratio would be measured. However, the molar ratio of W/V was ~95:5 (Figure S11) or 16:1 (Figure S12) from the XPS analyses, which

was close to the result obtained by the STEM image (~4 at%), further confirming the absence of V-based contaminations in the transferred 1T sample. In addition, only C-O bond at 532.4 eV (Figure S11d and S12d) or C=O bond at 533.9 eV (Figure S12d) were obtained and no V-O bond from vanadium oxidation (530.0 eV)[7] was observed from the O 1s peak, further excluding the possibility of $V_2O_3$ or $VO_2$ in the transferred 1T sample. The C-O/C=O bonds should be caused by the slightly oxidation of HOPG on the surface during the transferring process.

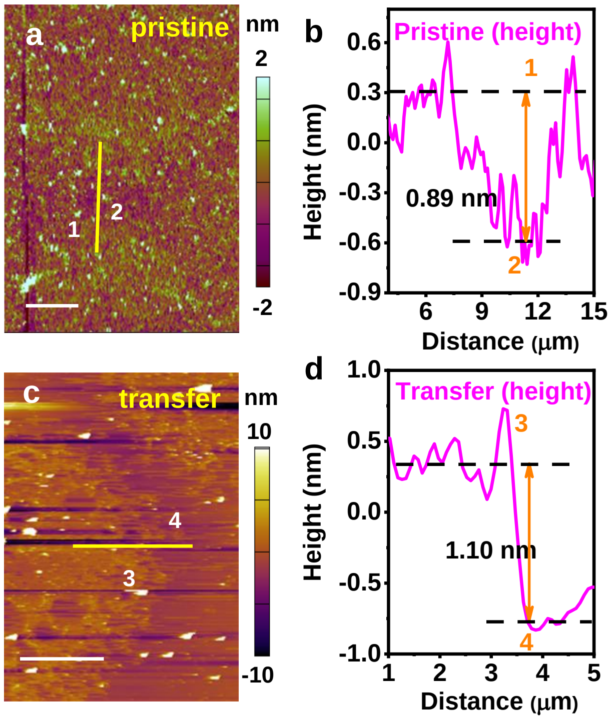


**Supplementary Fig. 13** AFM imaging of pristine and transferred 1T-$WS_2$. **a,** topographic image of pristine V SACs@1T-$WS_2$ on $V_2O_3$/sapphire; **b,** pristine height profile; **c,** topographic image of transferred V SACs@1T-$WS_2$ on sapphire; **d,** Transferred height profile. Scale bars: a, 5 μm; c, 5 μm.

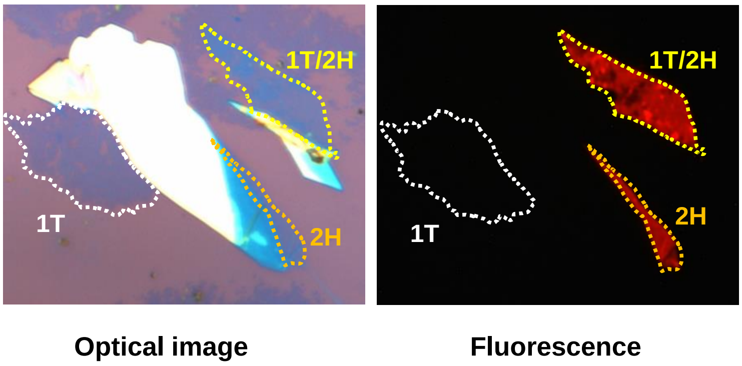


**Supplementary Fig. 14 Optical characterization of 1T and 2H phase.** Optical micrograph and fluorescence of V SACs@1T-$WS_2$ monolayer, 2H-$WS_2$ monolayer and trapezoidal 1T/2H bilayer heterostructure (formed by stacking of a 1T monolayer and a 2H monolayer). Fluorescence image was taken with a color camera, showing a dark emission state of V SACs@1T-$WS_2$, and bright emission state of 2H-$WS_2$ and 1T/2H bilayer heterostructure junction. The exposure time was 2000 ms.

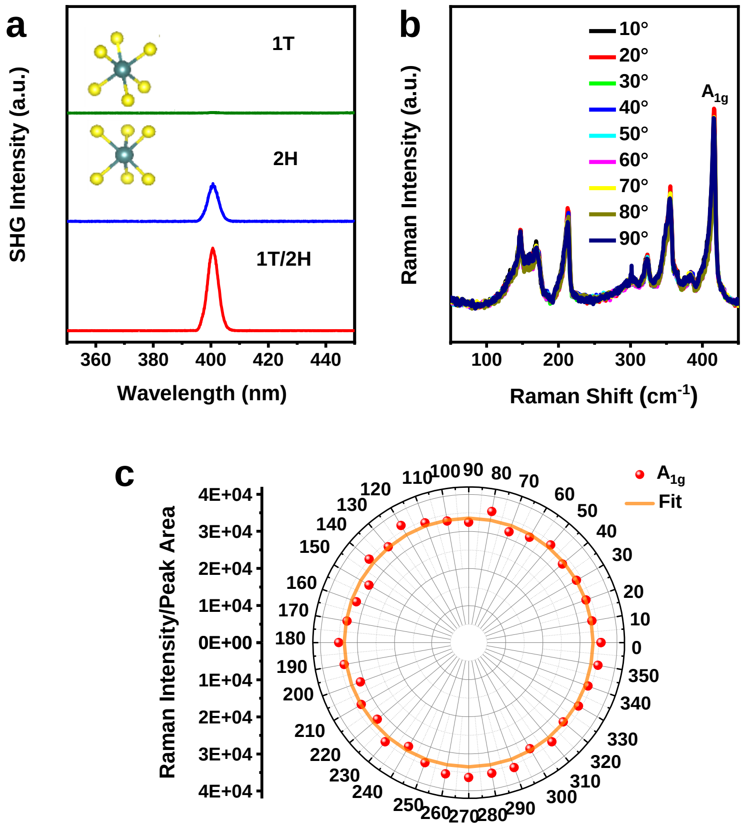


**Supplementary Fig. 15 Optical characterization of 1T and 2H phase. a,** SHG intensity collected from V SACs@1T-$WS_2$, 2H-$WS_2$ and bilayer 1T/2H heterostructure samples on $SiO_2$/Si substrates, respectively; **b,** Raman spectra of monolayer V SACs@1T-$WS_2$ as a function of rotating angle; **c,** Polarization Raman intensity from V SACs@1T-$WS_2$ as a function of rotating angle. The polar pattern of the $A_{1g}$ mode in the 1T phase was well fitted by a circle (orange solid line), which showed isotropic Raman scattering.

After transferring these samples (monolayer V SACs@1T-$WS_2$, monolayer 2H-$WS_2$ and bilayer 1T/2H heterostructure) on $SiO_2$/Si substrate, the underlying crystal symmetry could be obtained by means of SHG. As shown in Figure S14, three regions can be observed via optical and fluorescence microscope. The fluorescent image indicates that 1T/2H heterostructure is identical to 2H-$WS_2$ monolayer, suggesting non-coupling between 1T and 2H phases. More importantly, the 1T sample produces no SHG signal (Figure S15a green plot), matching well with an octahedral prismatic coordination ($D_{3d}$ group), in contrast to the evident SHG signals in the reported 1T' phase.[8-10] Meanwhile, a broken inversion symmetry of the 2H-$WS_2$ (blue plot) and 1T/2H (red plot) induces an intense SHG, because of the trigonal prismatic coordination in 2H-$WS_2$ samples ($D_{3h}$ group). The ARPRS of V SACs@1T-$WS_2$ was further performed by rotating laser polarization, as shown in Figure S15b. Moreover, Figure S15c proves the isotropic Raman scattering of V SACs@1T-$WS_2$, in which the polar pattern of the $A_{1g}$ mode (red dashed spheres) is well fitted by a circle (orange solid line), confirming the 1T features of as-grown $WS_2$. It is worth noting that the ARPRS of 1T'

TMD phase shows two-lobe pattern because of the non-centrosymmetric and anisotropic lattice structure[8,10].

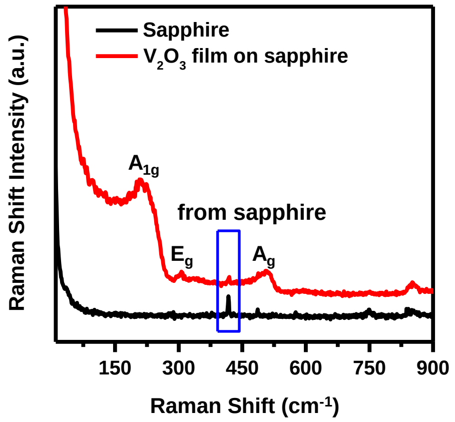


**Supplementary Fig. 16** Raman spectra of pure sapphire (black plot) and $V_2O_3$ film (red plot). The peak at 418.3 $cm^{-1}$ in the $V_2O_3$ film is derived from the $A_{1g}$ mode of the substrate sapphire[11]. The higher intensity merged peak in the range of 150~250 $cm^{-1}$ was correlation to both monoclinic $A_{1g}$ and hexagonal $V_2O_3$ $A_{1g}$ symmetry, indicative of a mixed phase. However, the additional peaks of low intensity at 300 $cm^{-1}$ and 500 $cm^{-1}$ were solely attributable to hexagonal $V_2O_3$[12,13], indicating the hexagonal structure of the as-grown $V_2O_3$ film.

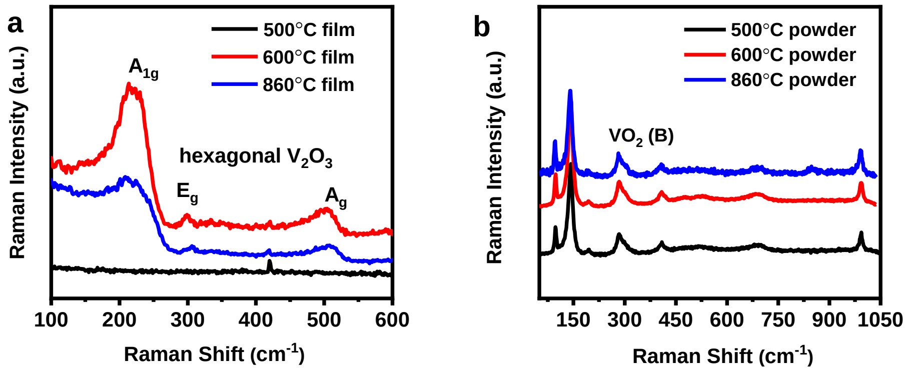


**Supplementary Fig. 17** **a**, The Raman spectra of $V_2O_3$ film formed under different grown temperatures. Notably, no $V_2O_3$ film has been formed at the temperature ≤ 500 °C; **b,** The Raman spectra of the powders in $VCl_3$ quart after reaction under different grown temperatures. From the Raman peaks positions, $VCl_3$ powder will firstly be decomposed as $VO_2$ (B)[14]. Then the $VO_2$ (B) will probably react with $H_2$ to form the final $V_2O_3$. We suggest the whole process for the $V_2O_3$ formation, as shown in the following equation (1) and (2).

$$2xVCl_3+4WO_3+3xH_2=2xVO_2\ (B)+4WO_{3-x}+6xHCl \quad (1)$$

$$2VO_2\ (B) + H_2 = V_2O_3 + H_2O \quad (2)$$

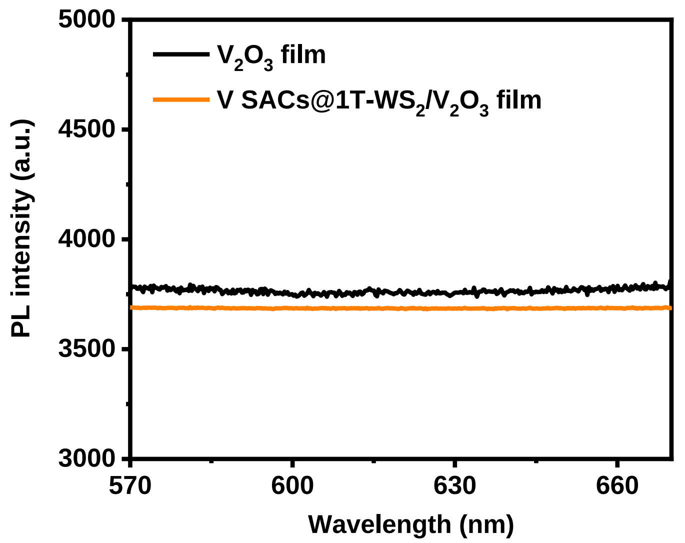


**Supplementary Fig. 18** Enlarged PL spectra of $V_2O_3$ film (black plot) and 1T-$WS_2$/$V_2O_3$ film (orange plot).

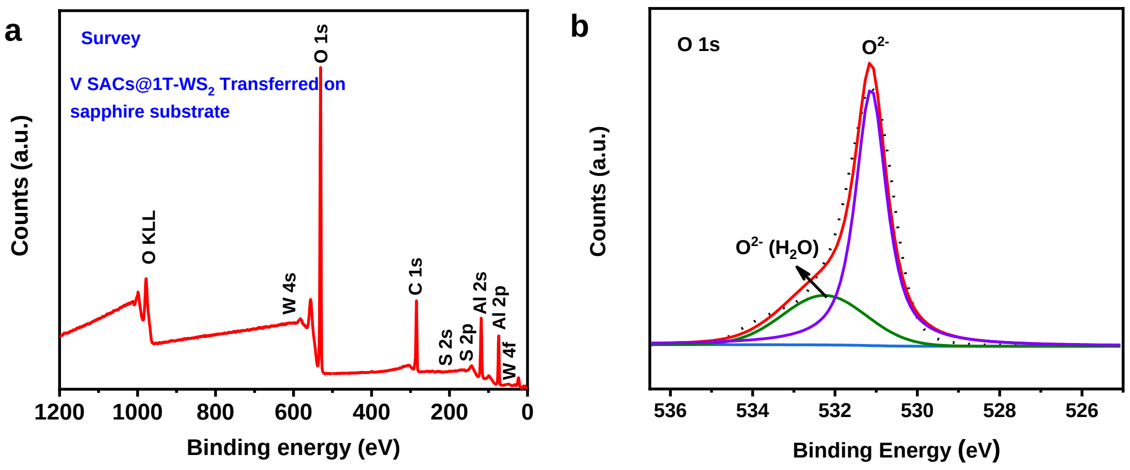


**Supplementary Fig. 19 a,** Survey scan of the V SACs@1T-$WS_2$ sample transferred on the sapphire substrate; **b,** High-resolution of XPS spectrum of O 1s.

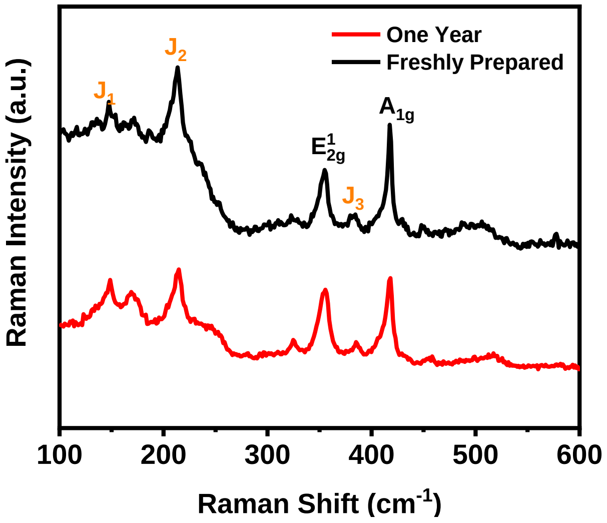


**Supplementary Fig. 20** Raman spectra of freshly prepared V SACs@1T-$WS_2$ sample on the $V_2O_3$ film (black plot) and after keeping for one year (red plot).

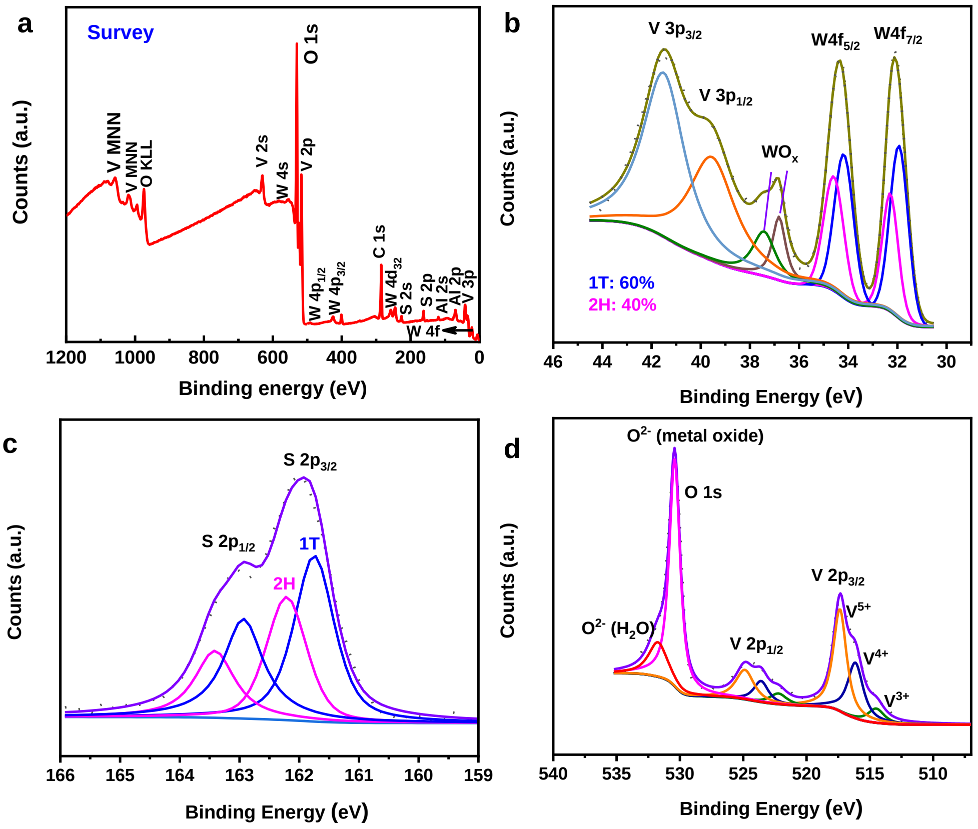


**Supplementary Fig. 21 a,** Survey spectra of V SACs@1T-$WS_2$/$V_2O_3$ film grown on sapphire substrate after 1 year; **b-d,** High-resolution XPS spectra of V 3p (b), W 4f (b), S 2p (c), O 1s (d) and V 2p (d) core level peak regions. The fitting blue and pink curves represent the contributions of 1T and 2H phases in Fig. S21b-c, respectively.

The wide-scan spectrum was shown in Fig. S21a and the main components are O, V, W, S, Al (from the sapphire). A high amount of 1T phase is still preserved even after

one year (~60%) from Fig. S21b, indicating the durable stability of 1T sample. Meanwhile, the V3p was found in Fig. S21b, which could not be used to analyze the V-base oxidation states caused by a complicated interaction between V3p and 3d electrons.[7] However, three contributions were found in the V2p XPS spectrum, as shown in Fig. S21d: $V^{3+}$, $V^{4+}$, $V^{5+}$, respectively, at 514.47 eV, 516.19 eV and 517.40 eV.[7] The main component is $V^{5+}$, which should be caused by the oxidation of $V_2O_3$ film on the surface in the air. The $V^{4+}$ signal from V-S bond in the 1T sample was also involved due to the overlapped peak position of $VS_2$ and $VO_2$. Please note that no $VO_2$ or $V_2O_5$ signals were detected by the Raman from Fig. S20 (red plot), which should be caused by overwhelming signals from 1T sample and $V_2O_3$ film on the surface of sample.

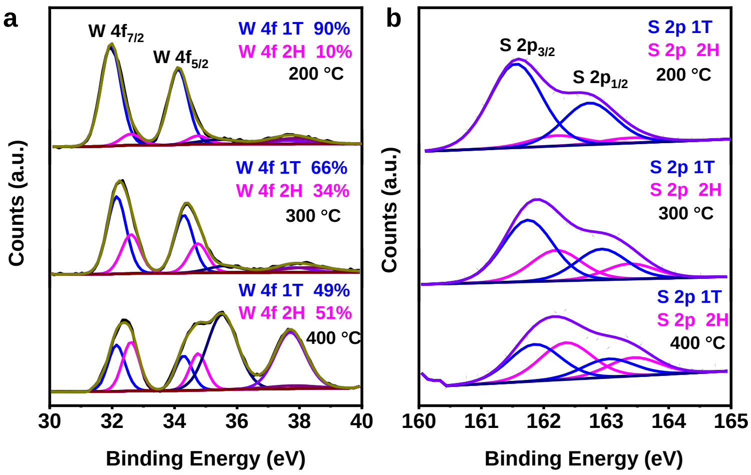


**Supplementary Fig. 22** High resolution XPS spectra of W 4f **(a)** and S 2s **(b)** core level peak regions for 1T-200 ºC, 1T-300 ºC, 1T-400 ºC samples. The fitting blue and pink curves represent the contributions of 1T and 2H phases, respectively. Please note that three pieces of 1T samples on the sapphire substrates were annealed at different temperatures in $H_2$/Ar for 2h.

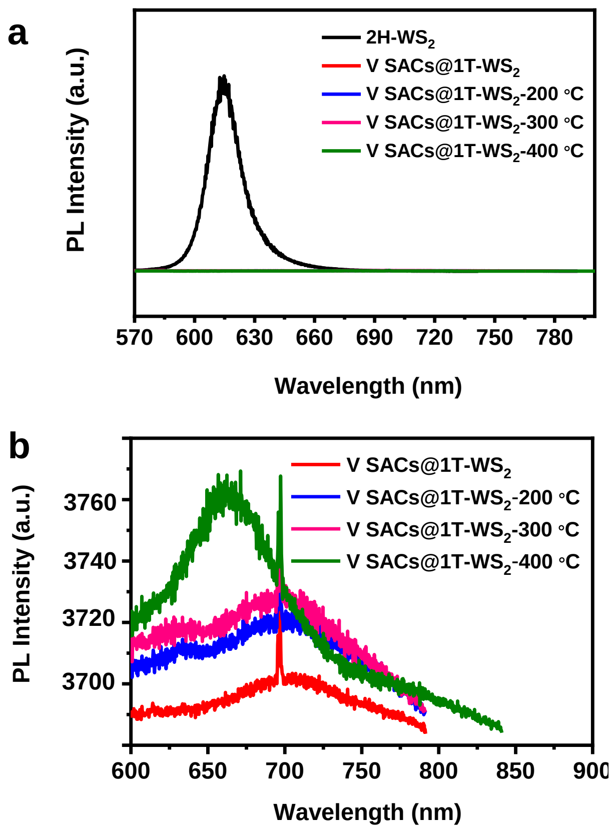


**Supplementary Fig. 23 a,** PL spectra of 2H-$WS_2$, V SACs@1T-$WS_2$, and V SACs@1T-$WS_2$ with different annealing temperatures in $H_2$/Ar for 2h; **b,** Enlarged PL spectra of V SACs@1T-$WS_2$ samples with different annealing temperatures. For all the V SACs@1T-$WS_2$ samples, the intensity of PL are almost completely suppressed, indicating the typical metallic behavior[15]. Meanwhile, compared with V SACs@1T-$WS_2$-200 ºC sample and V SACs@1T-$WS_2$-300 ºC sample, the PL intensity showed much higher intensity and blue shift in the V SACs@1T-$WS_2$-400 ºC sample, which may be attributed to band-structure modification due to the partial 1T phase transformation to 2H phase.

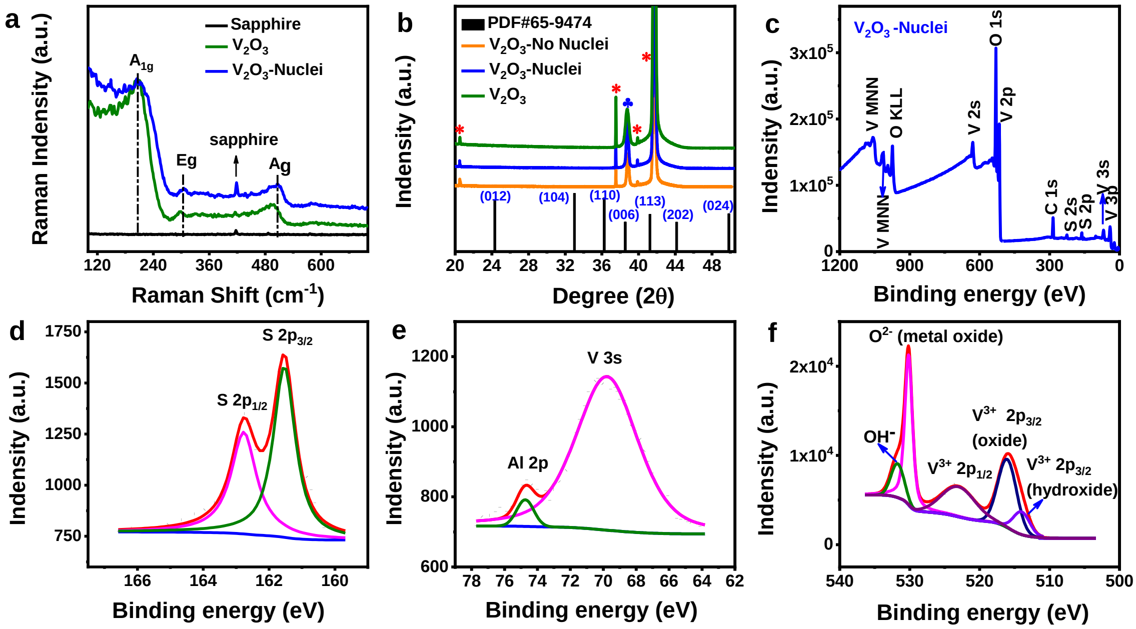

**Supplementary Fig. 24** **a**, Raman spectra of pure sapphire (black), $V_2O_3$ film (olive) and $V_2O_3$-Nuclei film (blue); **b**, XRD spectra of $V_2O_3$-Nuclei film (blue), $V_2O_3$-No Nuclei film (orange) and $V_2O_3$ film (olive); **c**, Survey spectra of $V_2O_3$-Nuclei film on sapphire substrate; **d-f**, High-resolution XPS spectra showing the S 2p (d), Al 2p (e) and V 2p (f) core levels for the $V_2O_3$-Nuclei film on the sapphire substrate sample. Please note that $V_2O_3$-No Nuclei film was prepared using $VCl_3$ precursor only. The oxygen was deduced from the residue oxygen in the tube furnace. The Raman spectrum of $V_2O_3$ film (olive) in Fig. S24a was measured on the 1T/$V_2O_3$ sample where no $WS_2$ appeared. The XRD pattern in Fig. S24b (olive) was obtained on the 1T/$V_2O_3$ sample.

The XRD analysis of different $V_2O_3$ films were conducted in the 2θ scanning mode, as shown in Figure S24b. All observed diffraction peaks have been indexed to the $V_2O_3$ (006),[16-18] demonstrating the epitaxial growth of the single-crystalline $V_2O_3$ film on sapphire substrate.

The high-resolution scans were made of V 2p, O1s, S 2p and Al 2p, as shown in Figure S24d-S24f, respectively. The $V2p_{3/2}$ peak was found to be at 514.15 eV (Figure S24f), the value falls within the range of 513.0-516.8 eV found in literature for $V_2O_3$.[19,20] Moreover, the $V2p_{3/2}$ peak at 516.12 eV was assigned to $V^{4+}$ from $VS_2$.[21,22] In addition, the O1s peaks showed very good agreement with literature for O peak associated with $V_2O_3$ absorbed OH ions or $H_2O$, the peaks of which were within the range of 530.3-530.5 eV found in literature.[19,23] The S 2p peaks at 161.6 eV and 162.7 eV can be indexed to S $2p_{3/2}$ and S $2p_{1/2}$ for $S^{2-}$, respectively, representing the $VS_2$ presence during the $V_2O_3$-Nuclei film deposition.[21,22,24]

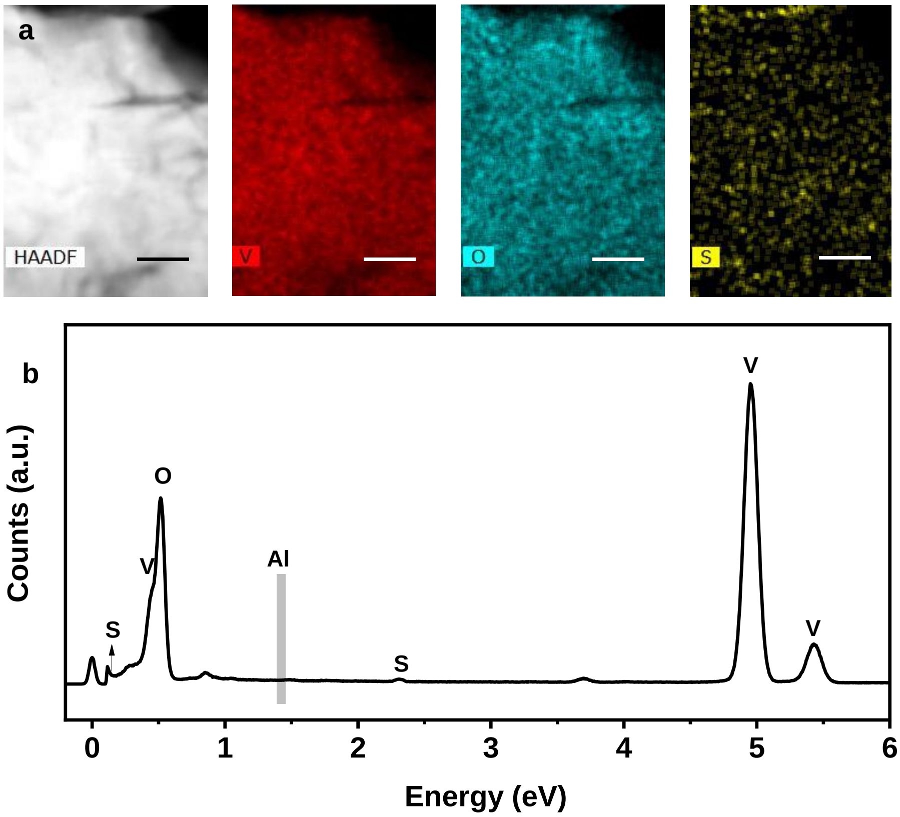


**Supplementary Fig. 25 a,** Elemental mapping of $V_2O_3$-Nuclei film. The film was scratched from sapphire substrate for HRTEM measurement; **b,** EDX spectrum of $V_2O_3$-Nuclei film. Scale bars: 40 nm.

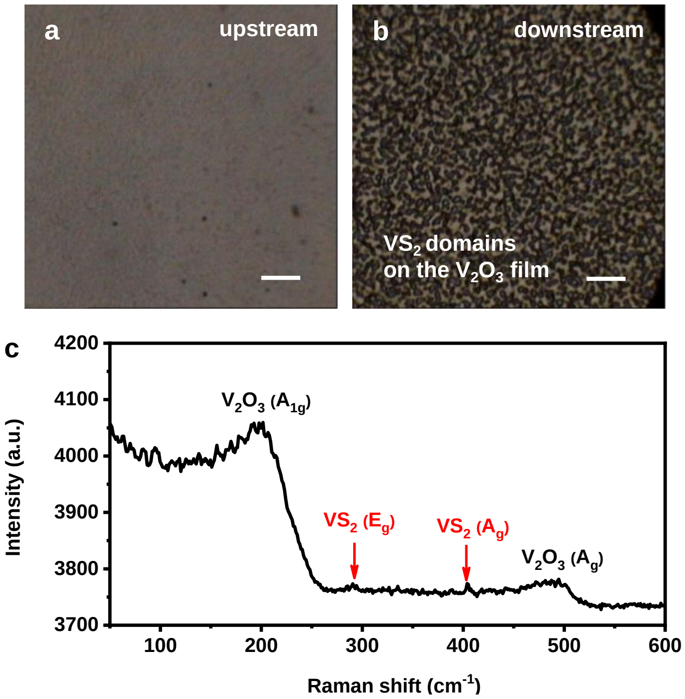


**Supplementary Fig. 26 a-b,** Optical micrographs of $V_2O_3$-Nuclei film in the upstream (a) and downstream (b); **c,** Raman spectrum of $V_2O_3$-Nuclei film in the downstream. The peak at 406.3 $cm^{-1}$ was assigned to $VS_2$ and different with the peak at 418.3 $cm^{-1}$, which was derived from the $A_{1g}$ mode of the substrate sapphire[11]. The recorded Raman peaks of $VS_2$ in Figure S26 were very weak, indicating that the amount of $VS_2$ was in very low concentration relative to $V_2O_3$. Scale bars: a-b, 90 μm.

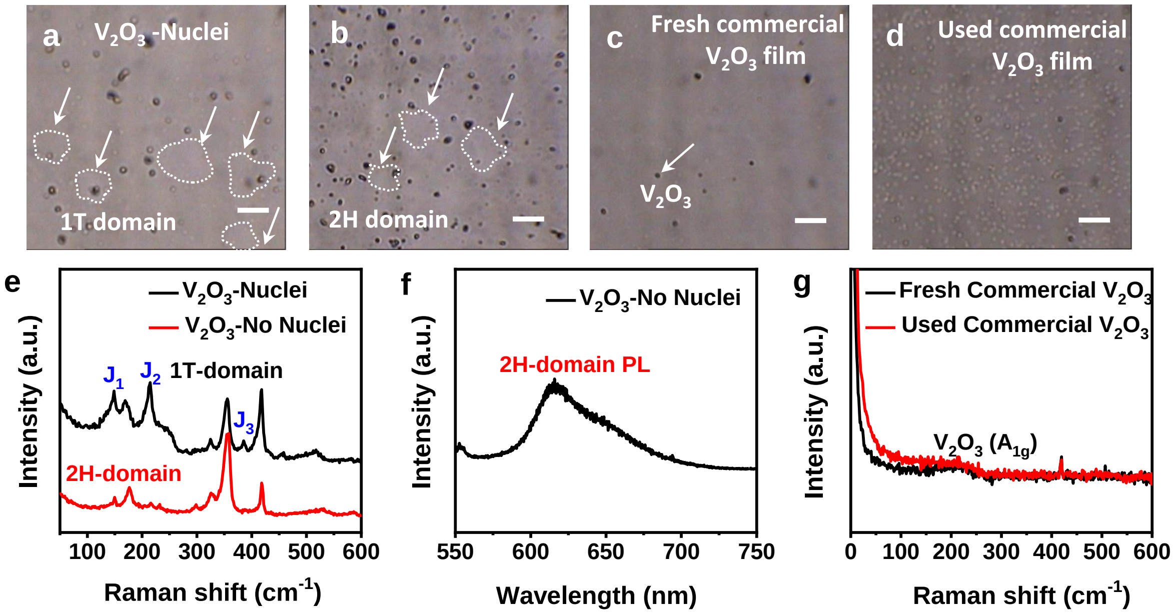


**Supplementary Fig. 27 a,** Optical micrograph of 1T domains formed on the $V_2O_3$-Nuclei film; **b,** Optical micrograph of 2H domains formed on the $V_2O_3$-No Nuclei film; **c,** Optical micrograph of fresh commercial $V_2O_3$ film; **d,** Optical micrograph of used commercial $V_2O_3$ film; **e,** Raman spectra of 1T domains formed on the $V_2O_3$-Nuclei film (black plot) and 2H domains formed on the $V_2O_3$-No Nuclei film (red plot); **f,** PL spectrum of 2H domains on the $V_2O_3$-No Nuclei film; **g,** Raman spectra of commercial $V_2O_3$ film before (black plot) and after (red plot) CVD growth. Scale bars: a-b, 10 µm.

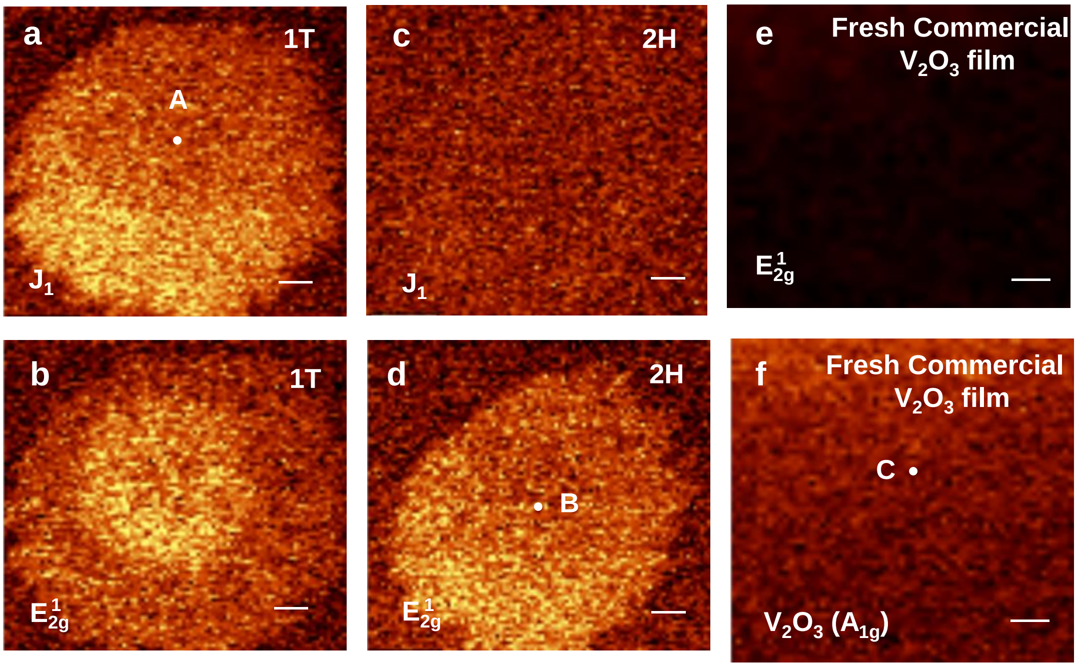


**Supplementary Fig. 28 a-b,** Raman mapping images of a 1T-$WS_2$ flake obtained in the $J_1$ (a) and $E^1_{2g}$ (b) vibrational modes, respectively. The measured $WS_2$ flake was grown on the $V_2O_3$-Nuclei film substrate; **c-d,** Raman mapping images of a 2H-$WS_2$ flake obtained in the $J_1$ (c) and $E^1_{2g}$ (d) vibrational modes, respectively. The measured $WS_2$ flake was grown on the $V_2O_3$-No Nuclei film substrate; **e-f,** Raman mapping image of the fresh commercial $V_2O_3$ film obtained in the $E^1_{2g}$ ($WS_2$) vibrational mode (e) and $A_{1g}$ ($V_2O_3$) mode (f). Scale bars: a-d, 1 μm; e-f, 5 μm.

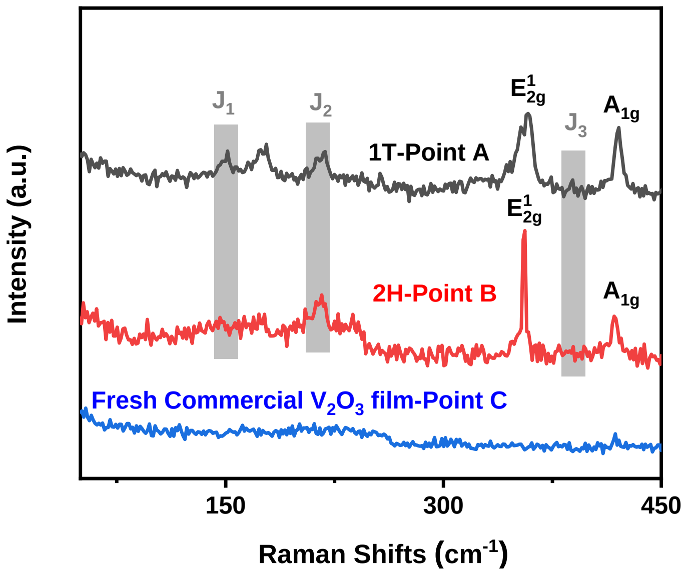


**Supplementary Fig. 29** Raman spectra of the selected three points (A, B, C) on their respective substrates in Supplementary Fig. 28a, 28d and 28f, respectively.

Raman mapping was directly conducted on the $WS_2$@$V_2O_3$ film to identify the 1T or 2H domains formed on the different $V_2O_3$ films, as shown in Supplementary Fig. 28. Fig. S28a-b and Fig. S28c-d are taken on 1T and 2H domain, respectively. Obviously, homogeneous signals from $J_1$ mode of 1T character were detected all over the domain (Fig. S28a), indicating the $WS_2$ domains grown on the $V_2O_3$-Nuclei film were metallic 1T phase. The corresponding Raman spectrum of point A was displayed in Supplementary Fig. 29 (black plot), showing clear $J_1$ peak which only belongs to 1T metallic phase. The signals of $E^1_{2g}$ resonance modes of $WS_2$ were observed on both the domains formed on $V_2O_3$-Nuclei film (Supplementary Fig. 28b) and $V_2O_3$-No Nuclei film (Supplementary Fig. 28d), respectively, confirming the formation of $WS_2$. However, no signals of $J_1$ mode were detected in the $WS_2$ domain formed on the $V_2O_3$-No Nuclei film, indicating that the $WS_2$ domain was 2H semiconducting phase. The typical Raman spectrum taken at point B in Supplementary Fig. 29 (red plot) clearly demonstrated the absence of $J_1$ peak, further confirming the 2H phase.

Moreover, as a contrast, the Raman mapping of fresh commercial $V_2O_3$ film was also carried out to identify the domains in Fig. S27c with different contrast. Apparently, no signals of $E^1_{2g}$ resonance modes of $WS_2$ were detected in Supplementary Fig. 28e, indicating no $WS_2$ was formed on the surface. However, the signals of $A_{1g}$ mode of $V_2O_3$ film was observed, demonstrating the $V_2O_3$ structure.

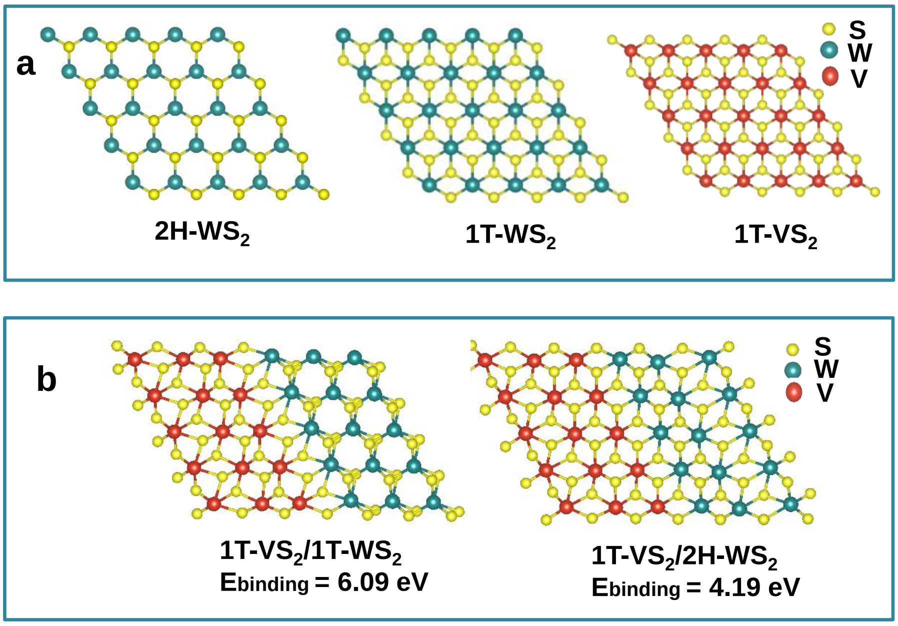


**Supplementary Fig. 30 a,** Schematic Structures of 2H-$WS_2$, 1T-$WS_2$ and 1T-$VS_2$, respectively; **b,** Binding energy of 1T-$VS_2$/1T-$WS_2$ and 1T-$VS_2$/2H-$WS_2$.

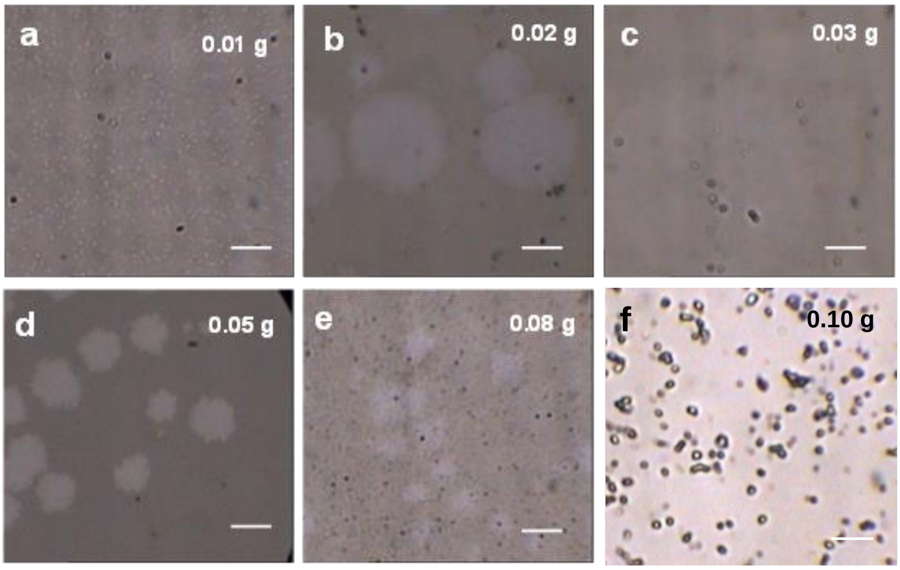


**Supplementary Fig. 31** Optical micrographs of as-prepared products using different amount of $VCl_3$. **a,** 0.01g; **b,** 0.02 g; **c,** 0.03 g; **d,** 0.05 g; **e,** 0.08 g; **f,** 0.10 g. $T_1$=860 °C, $T_2$ = 160 °C, Ar/$H_2$ = 80 sccm/20 sccm, t = 15 min; $T_1$ refers to the heating temperature of the furnace, $T_2$ refers to the heating temperature of the sulfur powder, and t refers to the growth time. All the other experimental parameters are the same. Scale bars: 5 μm.

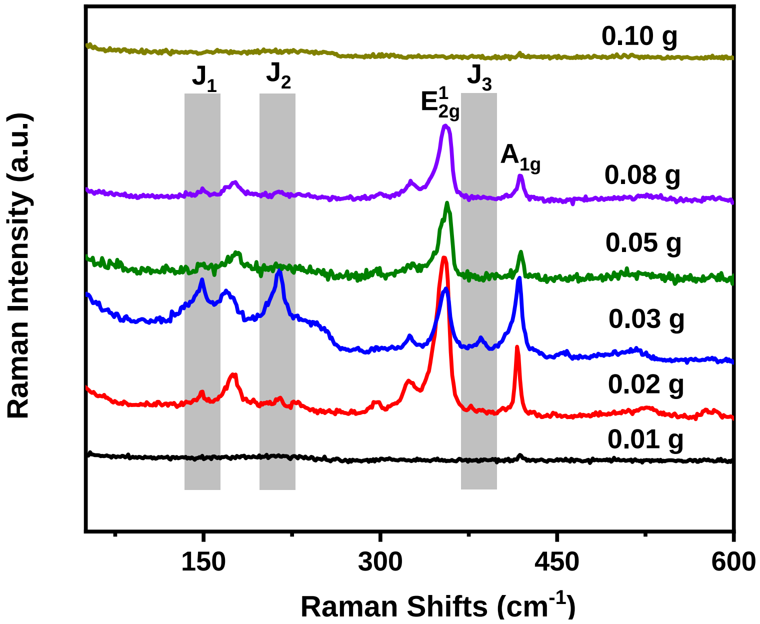


**Supplementary Fig. 32** The Raman spectra of as-prepared products using different amount of $VCl_3$.

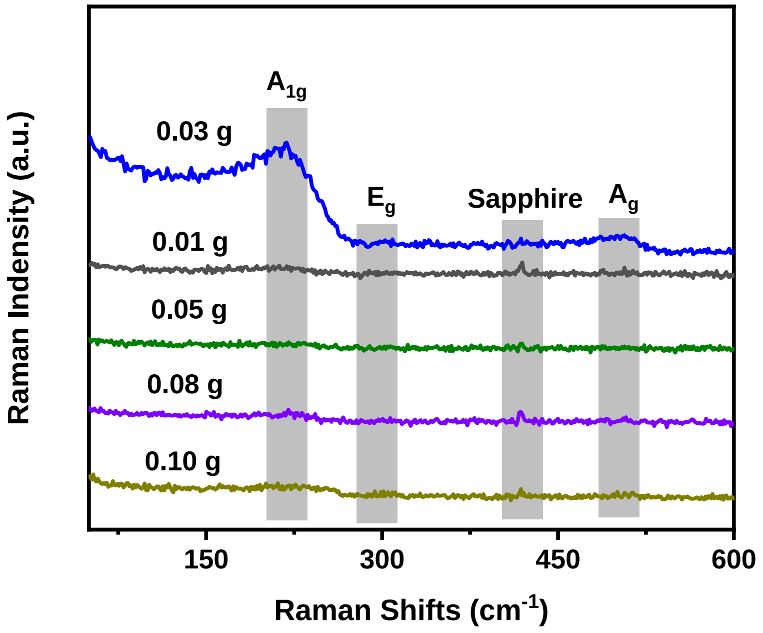


**Supplementary Fig. 33** The Raman spectra of as-prepared $V_2O_3$ film using different amount of $VCl_3$. The Raman spectra were measured on the areas where no $WS_2$ domains appeared.

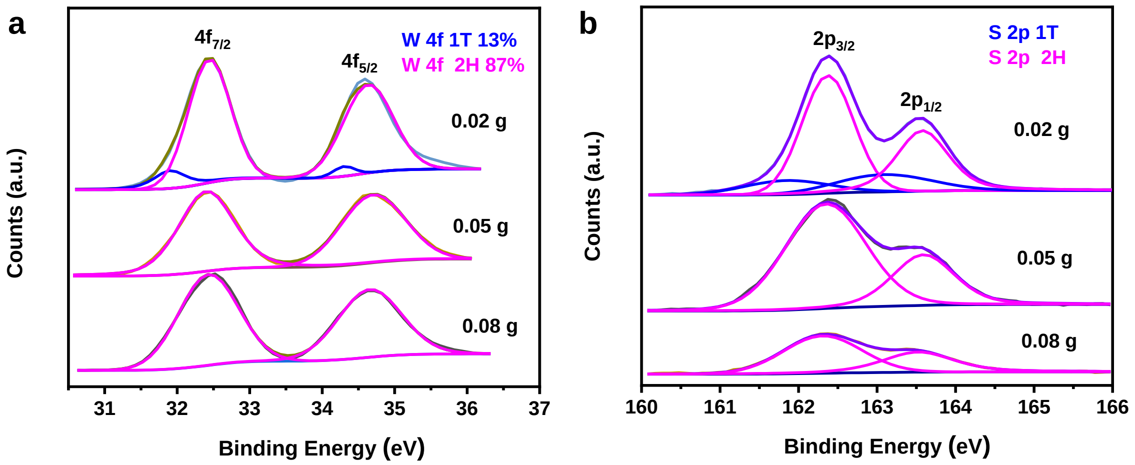


**Supplementary Fig. 34** High resolution XPS spectra of W 4f **(a)** and S 2s **(b)** core level peak regions for $WS_2$ domains using different amount of $VCl_3$. The fitting blue and pink curves represent the contributions of 1T and 2H phases, respectively. Please note that obtained $WS_2$ domains were transferred on the sapphire substrates for the XPS measurements.

In order to demonstrate the amount of $VCl_3$ has a great impact on the phase purity of $WS_2$, we have prepared $WS_2$ samples using different amount of $VCl_3$ (from 0.01 g to 0.10 g). The corresponding optical micrographs and Raman spectra were shown in

Supplementary Fig. 31-33. Apparently, when the amount of $VCl_3$ was 0.01 g, no $WS_2$ domains were formed on the sapphire substrate. As demonstrated in Supplementary Fig. 32 (black plot) and Supplementary Fig. 33 (black plot), the peaks that assigned to $V_2O_3$ were very weak, indicating the poor quality of $V_2O_3$ film.

In addition, when the amount of $VCl_3$ was increased to 0.02 g, dominated 2H-$WS_2$ samples were formed on the surface of $V_2O_3$ film. Particularly, the characteristic metallic peaks of $J_1$, $J_2$ and $J_3$ were gradually observed from Supplementary Fig. 32 (red plot). As demonstrated in the Fig. 4 in the revised manuscript, the 1T-$VS_2$ nuclei played important roles in determining the 1T-$WS_2$ phase growth. However, if the amount of $VCl_3$ was insufficient, it was deduced that only limited 1T-$VS_2$ nuclei were formed on the surface of $V_2O_3$ film to trigger the 1T-$WS_2$ growth. As a result, a low 1T/2H ratio of $WS_2$ were formed according to the XPS analysis (Supplementary Fig. 34).

Prominent of $J_1$, $J_2$ and $J_3$ peaks were clearly observed until the amount of $VCl_3$ was increased to 0.03 g, as shown in Supplementary Fig. 32 (blue plot). Simultaneously, high quality of $V_2O_3$ film was achieved if the $VCl_3$ amount was 0.03 g, as shown in Supplementary Fig. 33 (blue plot) and Supplementary Fig. 16 in the revised supplementary information. Appropriate formation of $VS_2$ nuclei and high quality of $V_2O_3$ were in favor of the epitaxial growth of 1T-$WS_2$ monolayers with high quality (Fig. 3c, 91% 1T-$WS_2$).

However, it was found than if the amount of $VCl_3$ was in the range of 0.05 g~0.08 g, no typical metallic peaks were observed from the Raman spectra in Supplementary Fig. 32 (olive and purple plots). Only 2H-$WS_2$ domains were formed, which were further corroborated by the XPS analyses (Supplementary Fig. 34). Moreover, it was believed that the large amount of $VCl_3$ could result in the high density of $VO_x$ vapor phase during the heating process, which could significantly accelerate the deposition rate of $V_2O_3$ film, leading to a $V_2O_3$ film with rough surface. Particularly, poor quality of $V_2O_3$ films were achieved when the amount of $VCl_3$ was in the range of 0.05g~0.08 g, as shown in Supplementary Fig. 33 (olive and purple plots). Moreover, no $WS_2$ domains were formed if the amount of $VCl_3$ was ~0.1 g due to the extremely rough surface of $V_2O_3$ film. From the analysis of the influence of amount of $VCl_3$ on the purity of 1T-$WS_2$, we can conclude that the quality of $V_2O_3$ film was the constraining factor to determine the formation of 1T-$WS_2$ with high phase purity when the amount of $VCl_3$ was higher than 0.03 g. Whereas, the density of $VS_2$ nuclei was the constraining factor to impact the 1T-$WS_2$ formation when the amount of $VCl_3$ was less than 0.03 g.

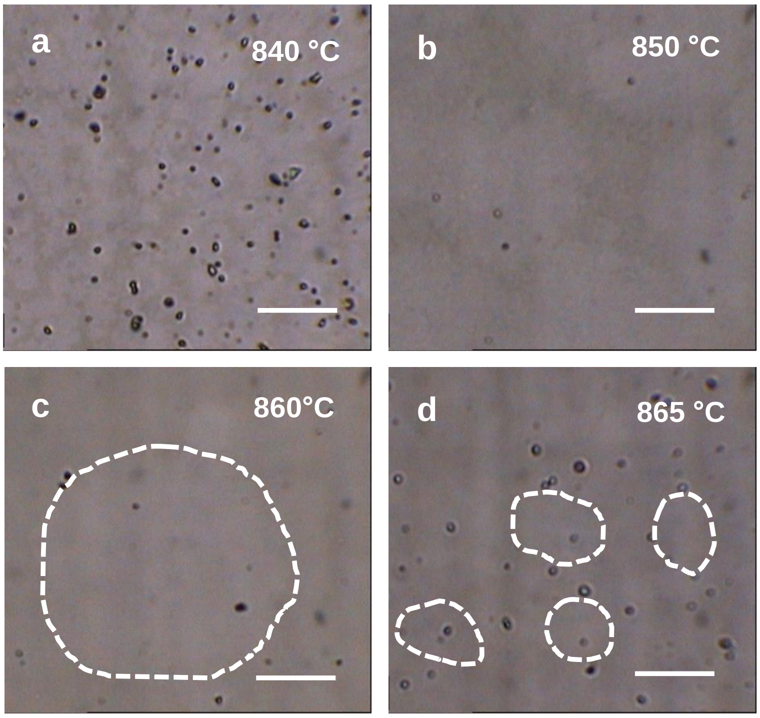


**Supplementary Fig. 35** 1T-$WS_2$ domains prepared at different heating temperatures ($T_1$). **a,** $T_1$=840 °C; **b,** $T_1$=850 °C; **c,** $T_1$=860 °C; **d,** $T_1$=865 °C. $T_1$ refers to the heating temperature of the furnace, $T_2$ refers to the heating temperature of the sulfur powder, and t refers to the growth time. All the other experimental parameters are the same except $T_1$. $T_2$ = 160 °C, Ar/$H_2$ = 80 sccm/20sccm, t = 15 min. Scale bars: 10 µm.

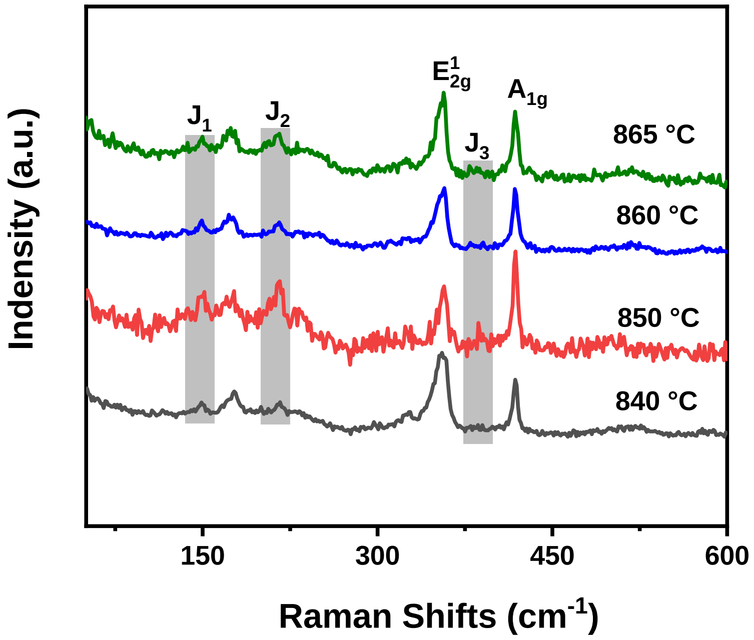


**Supplementary Fig. 36** The Raman spectra of $WS_2$ domain prepared at different heating temperatures of furnace.

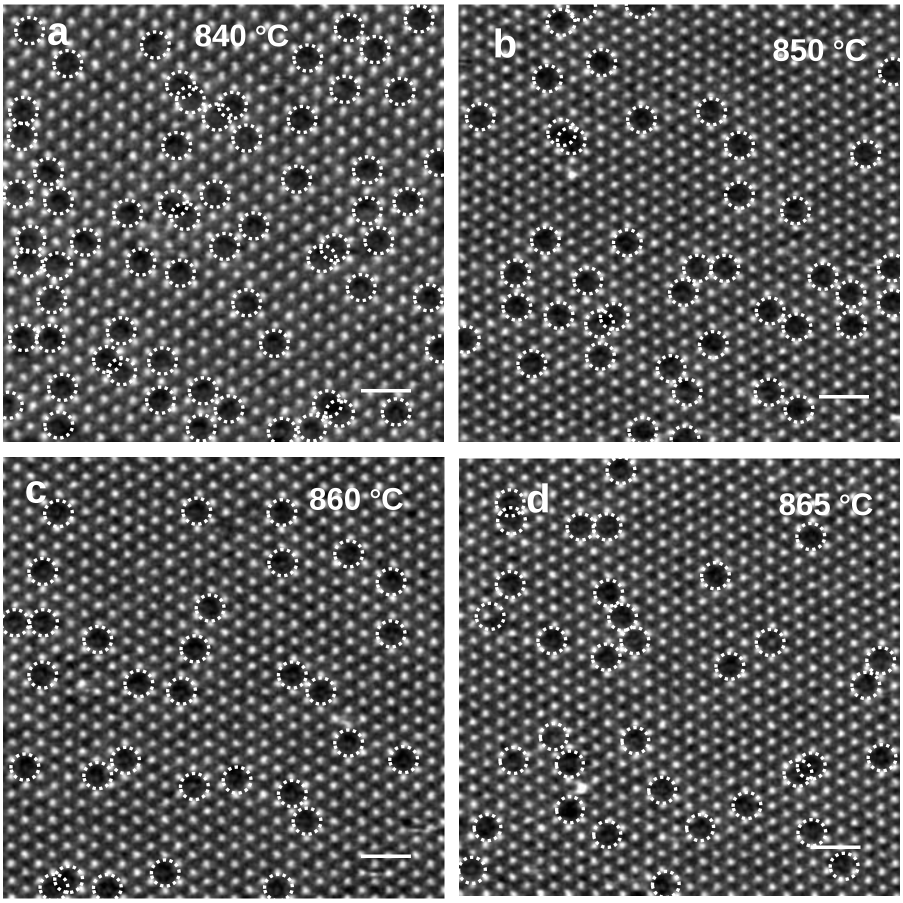


**Supplementary Fig. 37** STEM images of V SACs@1T-$WS_2$ monolayers prepared at different heating temperatures. Single V atoms are marked by the white dashed circles. Scale bars: 1 nm.

As expected, the $WS_2$ samples with different lateral sizes were formed under the different heating temperatures in Supplementary Fig. 35. Impressively, the typical metallic peaks ($J_1$, $J_2$, $J_3$) were all observed in the Raman spectra of Supplementary Fig. 36, indicative of the formation of 1T-$WS_2$. Moreover, the atomic structures of corresponding V SACs@1T-$WS_2$ samples were shown in Supplementary Fig. 37, which manifested the 1T structures of $WS_2$. In particular, the single V atoms were 8.0 at% (4.0 wt%), 5.0 at% (2.5 wt%), 4.0 at% (2.0 wt%) and 4.2 at% (2.1 wt%), respectively, when changing the heating temperatures from 840 °C to 865 °C, revealing that the V concentration in the 1T-$WS_2$ monolayers could be tuned by the heating temperature.

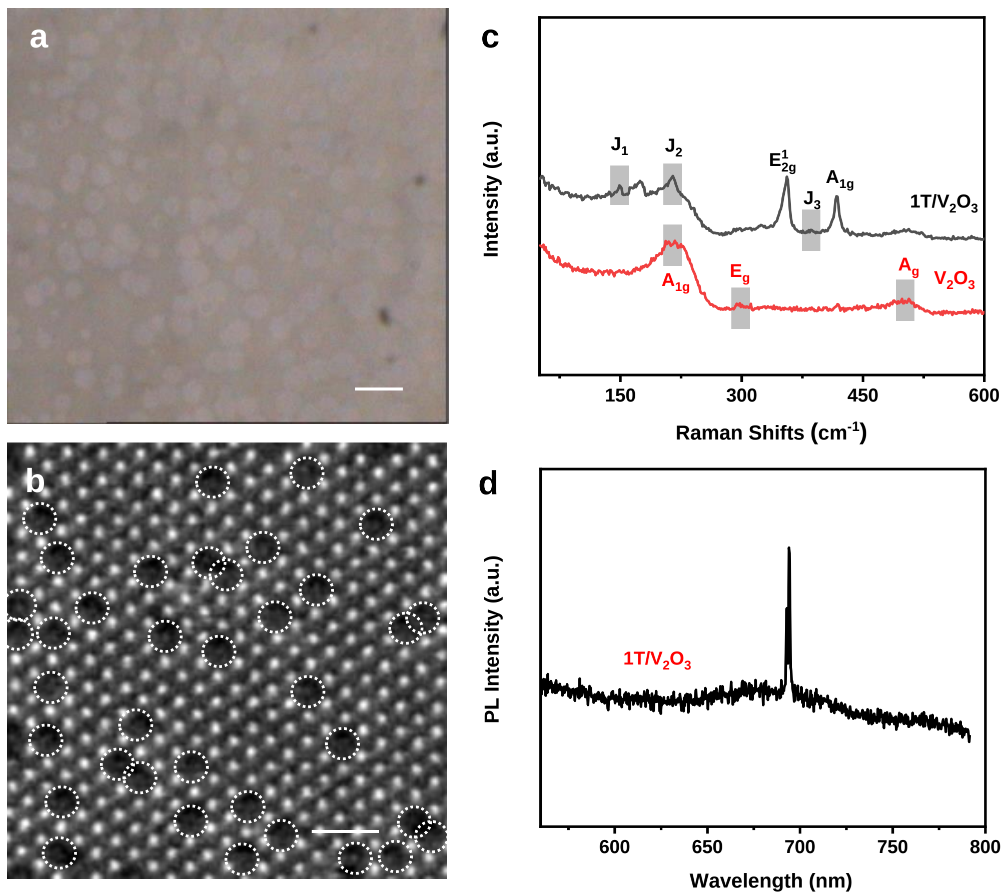


**Supplementary Fig. 38 a,** Optical micrograph of 1T-$WS_2$ domains obtained using vanadocene (0.08 g) as the co-precursors. $T_1$=860 °C, $T_2$ = 160 °C, Ar/$H_2$ = 80 sccm/20 sccm, t = 15 min; $T_1$ refers to the heating temperature of the furnace, $T_2$ refers to the heating temperature of the sulfur powder, and t refers to the growth time; **b,** STEM image of obtained V SACs@1T-$WS_2$ monolayer. The single V atoms are marked by the white dashed circles; **c,** Raman spectra of 1T/$V_2O_3$ (black plot) and $V_2O_3$ film (red plot), respectively; **d,** PL spectrum of 1T/$V_2O_3$ film. Scale bars: a, 20 µm; b, 1 nm.

Apparently, 1T-$WS_2$ domains were also achieved if using vanadocene as the co-precursors. However, it required higher amount of vanadocene (0.08 g) to obtain the 1T-$WS_2$ domains. The characterizations of V SACs@1T-$WS_2$ monolayers were shown in Supplementary Fig. 38. From the optical micrograph, the obtained 1T-$WS_2$ monolayers showed similar morphology with Fig. 2b. The atomic structure of V SACs@1T-$WS_2$ was displayed in Supplementary Fig. 38b, confirming the 1T phase of $WS_2$. The typical metallic peaks were prominently seen in the Raman spectrum (Supplementary Fig. 38c), indicative of the metallic feature of $WS_2$ domains. Moreover, the negligible PL intensity of obtained $WS_2$ domains was also provided to demonstrate the metallic nature (Supplementary Fig. 38d). It was reported that vanadocene could be completely decomposed at less than 200 °C under a $H_2$ atmosphere,[25] so it was easier to transport in the gaseous state than $VCl_3$ during the heating temperature. As a result, it required higher amount of vanadocene to obtain the 1T-$WS_2$ domains.

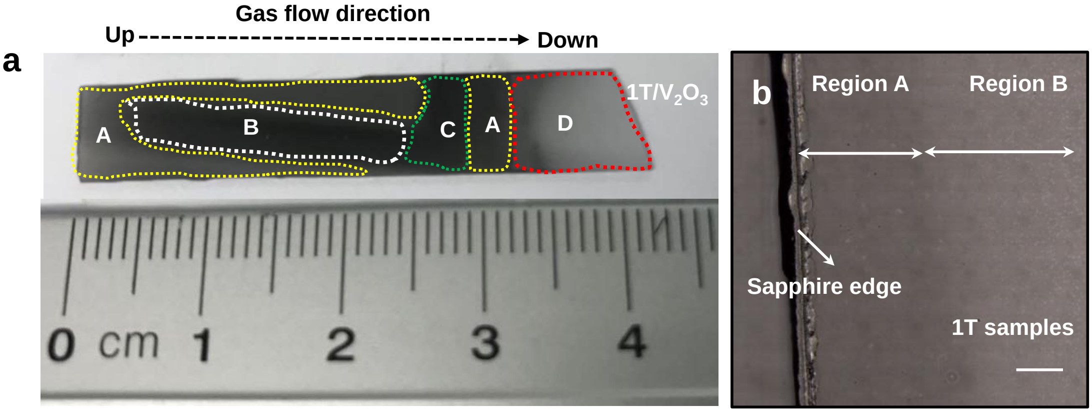


**Supplementary Fig. 39 a,** Picture of $1T/V_2O_3$ film grown on sapphire substrate with different growth regions. Region A represents the $V_2O_3$ film without 1T sample. Represent B represents the $V_2O_3$ film with isolated 1T flakes. Region D represents the very downstream area, which was not able to grow $V_2O_3$ film and $WS_2$ sample on the surface; **b,** Stitching optical micrograph of the interface between region A and region B. Please note that region B and region C are the main products, stitching optical micrograph was carried out for the clear observation of the morphology (see Fig. S40). Scale bar: b, 300 μm.

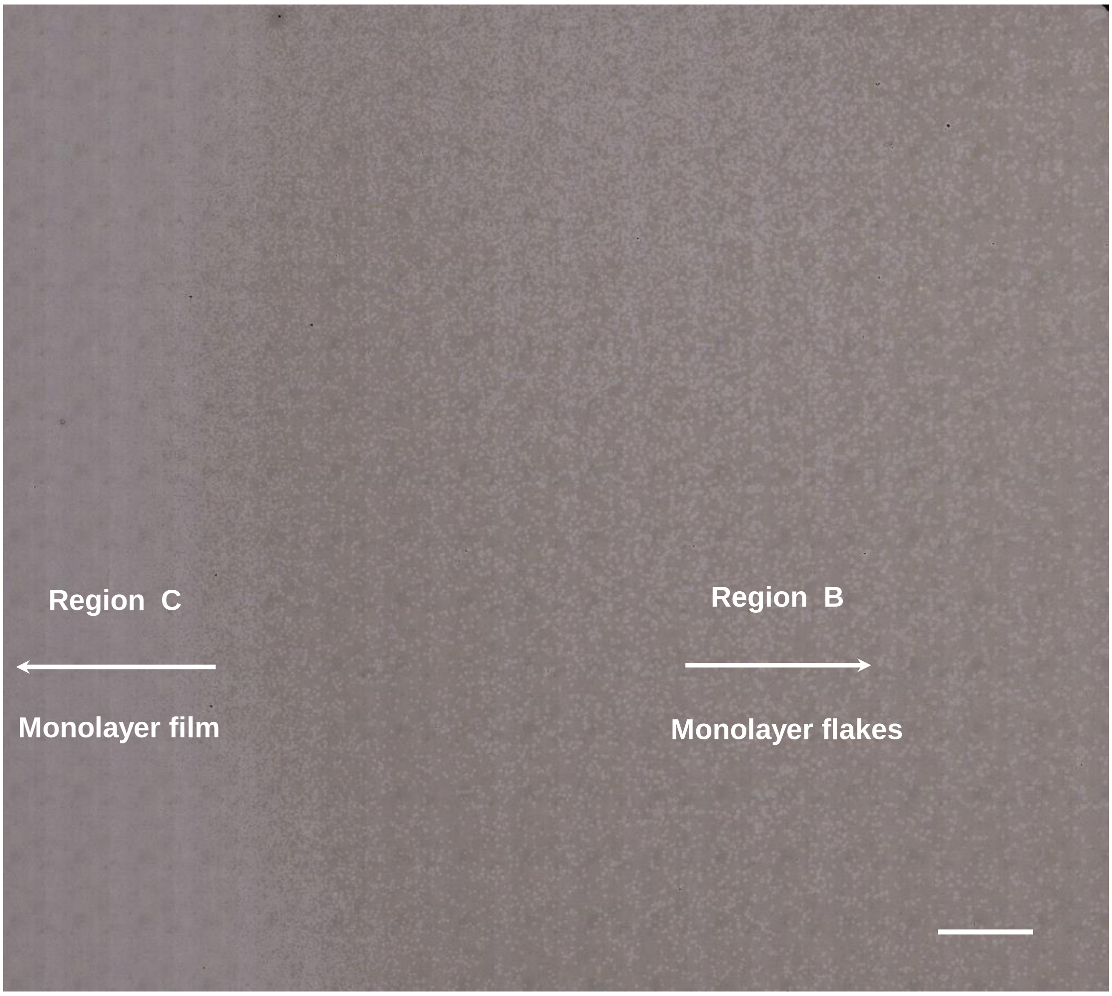


**Supplementary Fig. 40** Stitching optical micrograph of as-grown 1T-$WS_2$ monolayers on the surface of $V_2O_3$/sapphire. Please note that the stitching area was selected from the interface between region B and Region C in the Fig. S39a. Scale bar: 500 μm.

The isolated flakes (right white arrow) in growth region B have domain sizes from 20~30 μm. On the left side in growth region C, the $WS_2$ domains are merged into a continuous film. This kind of non-uniformity in nucleation density and domain size is a limitation of the present CVD technique, which have been demonstrated in many previously reported literatures about TMDs grown on Si/$SiO_2$[26,27], quartz[28] and sapphire substrates[29-31]. As a result, it is inevitable to grow isolated and merged film domains in our CVD process for 1T monolayers growth. However, each growth contained ~0.5 cm x 2.0 cm (~1.0 $cm^2$) region where hundreds of isolated flakes grown and ~0.5 cm x 1.0 cm (~0.5 $cm^2$) region where merged 1T film grown.

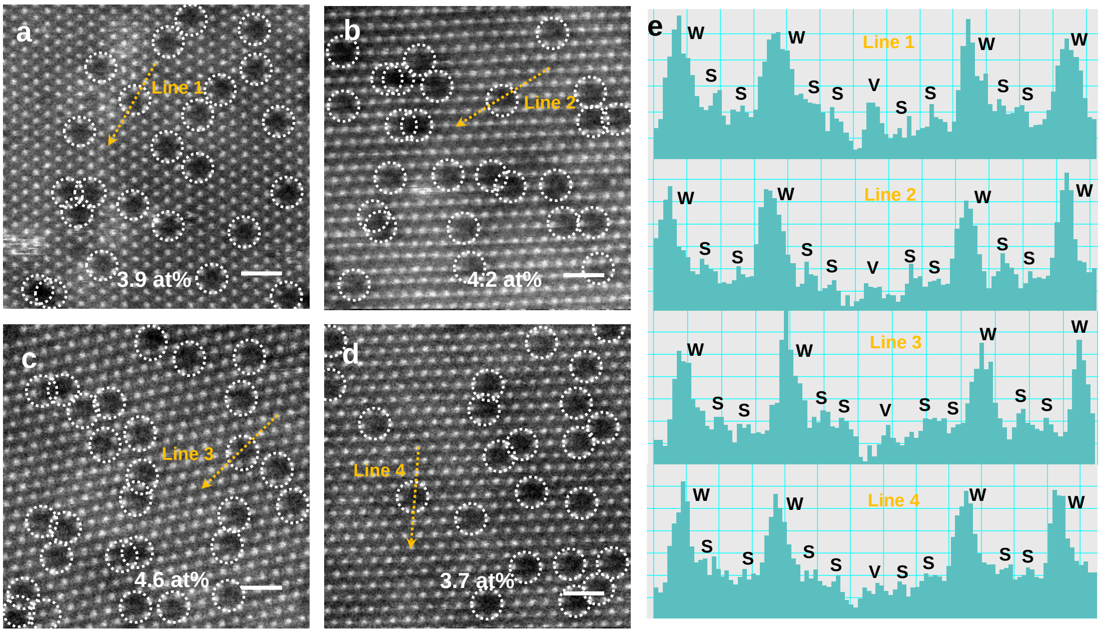


**Supplementary Fig. 41 a-d,** STEM images of V SACs@1T-$WS_2$ merged film grown in region C. Single V atoms are marked by the white dashed circles. The STEM images were randomly taken from six different areas; **e,** Intensity sequence profiles of W-S-S-V-S-S-W (orange dashed arrow indicated by Figure S41a-d). Scale bars: 1 nm.

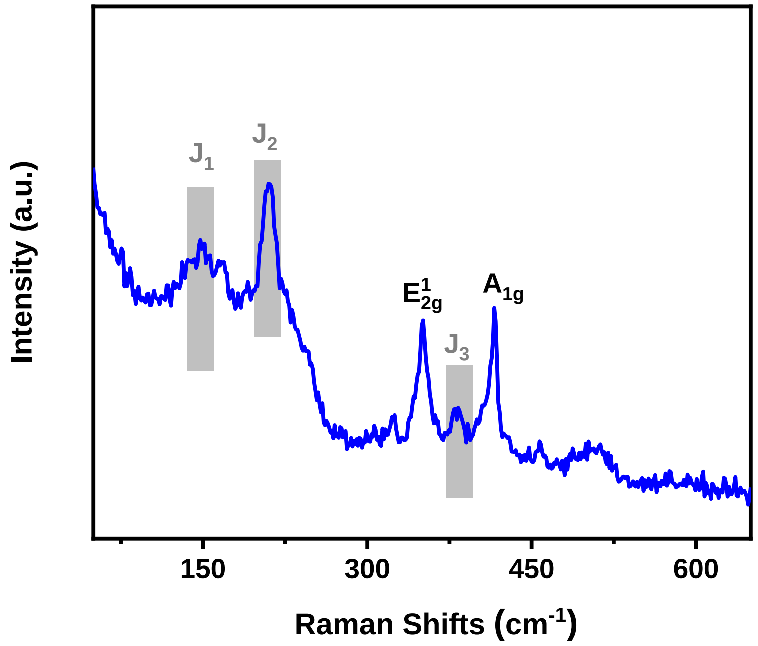


**Supplementary Fig. 42** Raman spectrum of 1T merged film/$V_2O_3$ in growth region C.

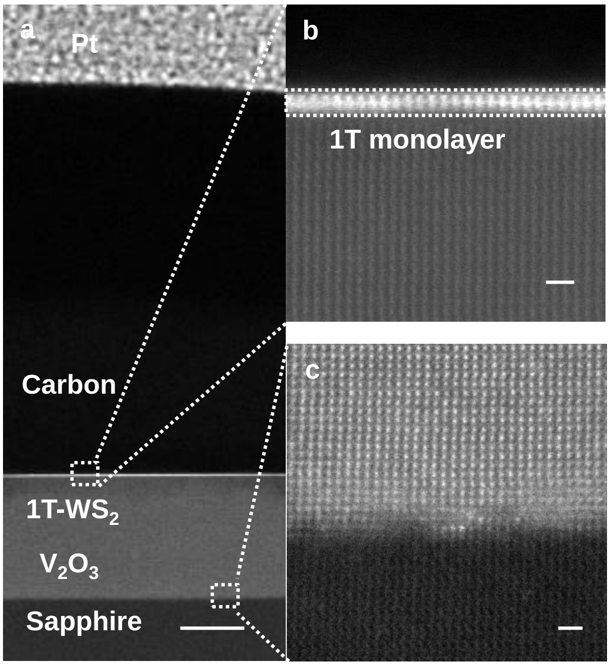


**Supplementary Fig. 43 a,** Low-resolution cross-section HAADF-STEM image of V SACs@1T-$WS_2$ merged film on the surface of $V_2O_3$ film/sapphire substrate; **b,** High-resolution HAADF-STEM image of V SACs@1T-$WS_2$ monolayer film on the $V_2O_3$ surface; **c,** High-resolution HAADF-STEM image of the interface between $V_2O_3$ and sapphire substrate. Scale bars: a, 10 nm; b-c, 0.5 nm.

The characterizations of V SACs@1T-$WS_2$ merged film in growth region C were shown in Supplementary Fig. 41-43. The atomic structure of V SACs@1T-$WS_2$ merged film were shown in Fig. S41. The images were randomly taken from four different areas in region C. The 1T phase of $WS_2$ was confirmed with V atoms distribution from the high-resolution STEM images. In particular, the average single V atoms was ~4.1 at% (2.1 wt%), which was close to the atomic density of V atoms in the growth region B (Fig. 2c, ~2.0 wt%). The W-S-S-V-S-S-W intensity profile sequences were also achieved to identify the V atoms replacement at W sites, as shown in Fig. S41e. The typical metallic peaks were shown in the Raman spectrum (Supplementary Fig. 42), confirming the metallic feature of $WS_2$ sample. The monolayer thickness was confirmed by cross-sectional STEM images in Fig. S43.

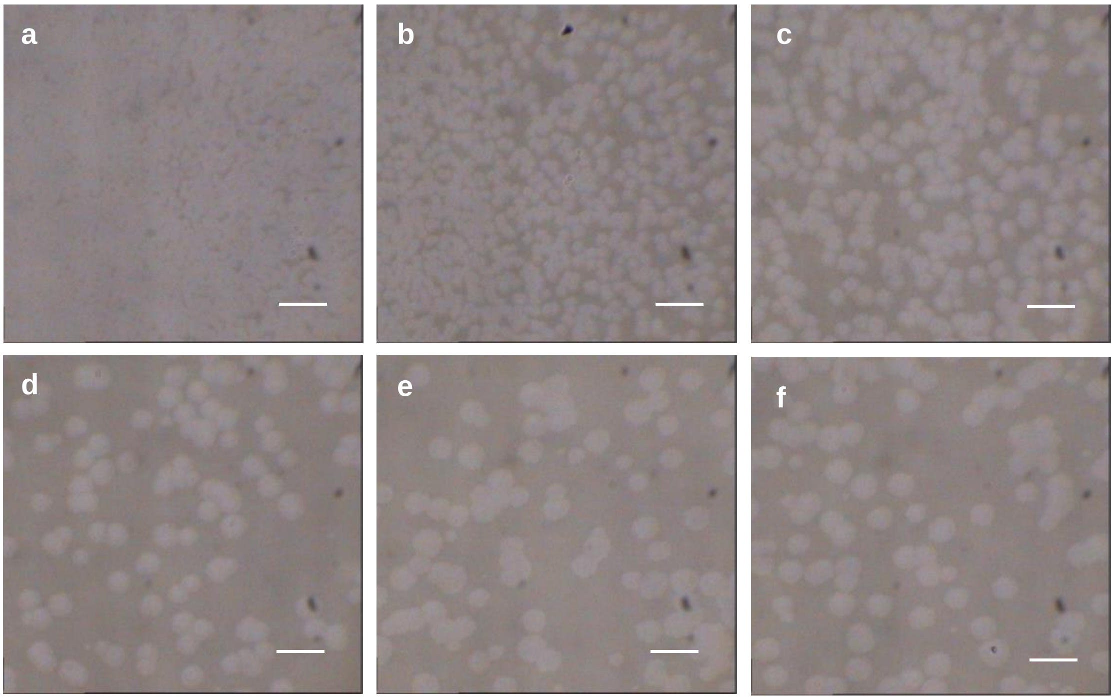


**Supplementary Fig. 44** Six representative optical micrographs taken from Fig. S40. Scale bars: 50 μm. To transfer the 1T samples for the investigation of HER catalytic performance, we transferred the samples at the region B (near the interface between region B and C). However, it was inevitable to transfer a small part of 1T merged film onto the glassy carbon electrode. To acquire a relatively accurate geometric area, we made statistics for the real geometric area of 1T samples. Please see details in Fig. S45 and Table S4-S6

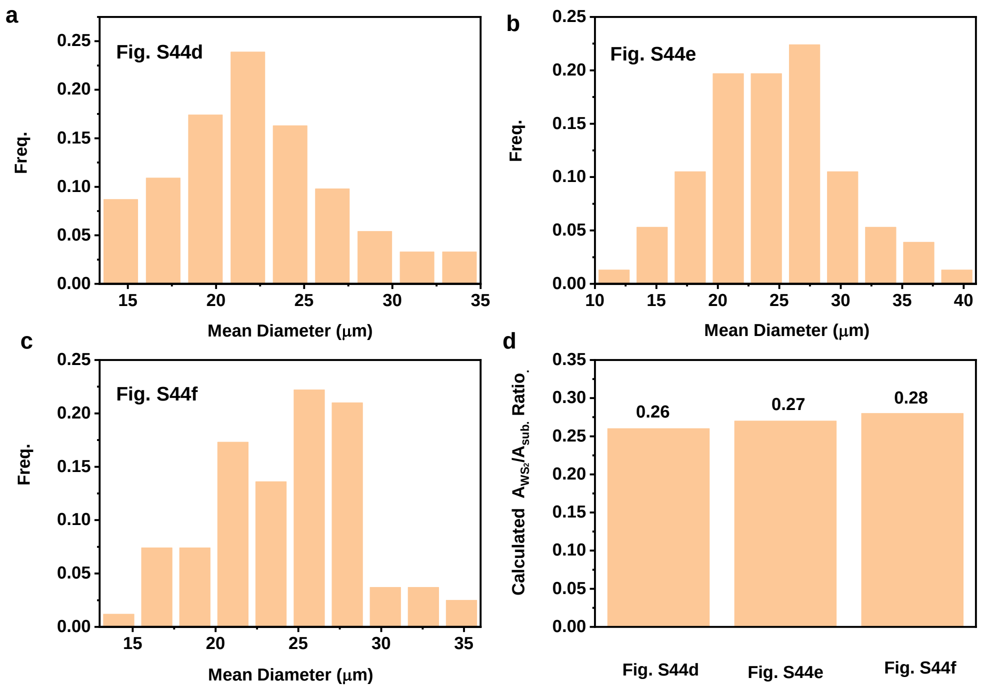


**Supplementary Fig. 45 a-c,** Statistics of $WS_2$ distribution with different lateral sizes in Fig. S44d (a), Fig. S44e (b) and Fig. S44f (c), respectively. **d,** the calculated $A_{WS_2}/A_{sub.}$ ratio in the respective Figs.

In order to evaluate the actual area of $WS_2$ monolayers more accurately, we made statistics for the actual area of 1T-$WS_2$ monolayers in Fig. S44, which was taken from stitching optical images in Fig. S40. $A_{WS_2}$represents the area of $WS_2$ monolayers in the respective picture. $A_{sub.}$ represents the area of respective picture. The stitching optical micrograph of $WS_2$ indicated that the as-grown $WS_2$ monolayers included both continuous film and isolated $WS_2$ monolayers. The continuous film was formed on the relatively down-stream of isolated $WS_2$ monolayers. Six representative areas were selected for the statistical analyses in Fig. S44. The largest $A_{WS_2}/A_{sub.}$ ratio in these Figs was ~1.0 from Fig. S44a due to the approximately continuous film. As for the Fig. S44b-44c, the ratio of $A_{WS_2}/A_{sub.}$ was apparently less than 1.0. Compared with Fig. S44a-S44c, the relatively lower ratios of $A_{WS_2}/A_{sub.}$ were shown in Fig. S44d-S44f according to the lower distribution density of $WS_2$ monolayers. To obtain the accurate $A_{WS_2}/A_{sub.}$ ratios of Fig. S44d-44e, the diameter distribution range of $WS_2$ monolayers was obtained by average diameter analysis software. The corresponding distribution sizes of $WS_2$ monolayers were displayed in Fig. S45a-S45c. According to the calculated area results in Table S4-S6. The $A_{WS_2}/A_{sub.}$ ratios were 0.26, 0.27 and 0.28, respectively. We take the mean value of 0.27 as the lowest $A_{WS_2}/A_{sub.}$ ratio. Consequently, the actual mass loading of $WS_2$ monolayers was in the range of 1.8~6.5 $\mu g/cm^2$.

The mass loading of continues $WS_2$ film was calculated according to the equation as follows:

$$Mass\ loading\ of\ WS_2 = \frac{m_{WS_2}}{A_{sub.}} = \frac{\rho_{WS_2} V_{WS_2}}{A_{sub.}} = \frac{\rho_{WS_2} A_{WS_2} H_{WS_2}}{A_{sub.}} = \rho_{WS_2} H_{WS_2}$$

$m_{WS_2}$, $\rho_{WS_2}$, $V_{WS_2}$, $H_{WS_2}$ represent the mass amount of $WS_2$, density of $WS_2$, volume of $WS_2$, and monolayer thickness of $WS_2$, respectively.

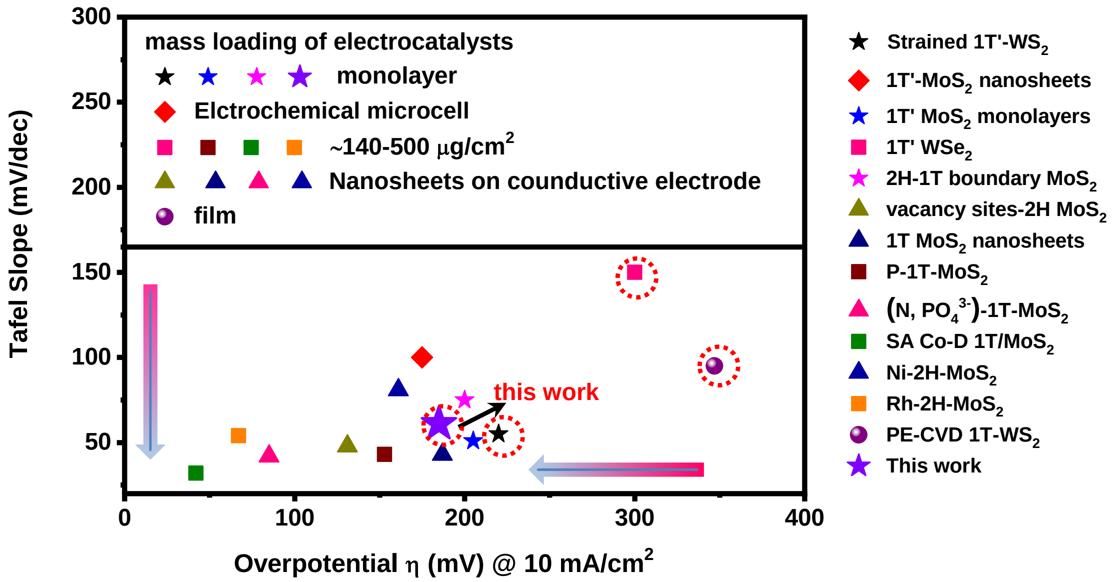


**Supplementary Fig. 46** HER performance comparison for the various catalysts with different mass loadings (the catalysts include strained 1T'-$WS_2$,[32] 1T'-$MoS_2$ nanosheets,[33] 1T'-$MoS_2$ monolayers,[9] 1T' $WSe_2$,[34] 2H-1T boundary $MoS_2$,[35] vacancy sites-2H $MoS_2$,[36] 1T-$MoS_2$ nanosheets,[37] P-1T-$MoS_2$,[38] (N,$PO_4^{3-}$)-1T-$MoS_2$,[39] SA Co-D-1T-$MoS_2$,[40] SA Ni-2H-$MoS_2$,[41] SA Rh-2H-$MoS_2$,[42] PE-CVD 1T-$WS_2$[43]). The red dashed circles represent the 1T(1T')-W based TMDs.

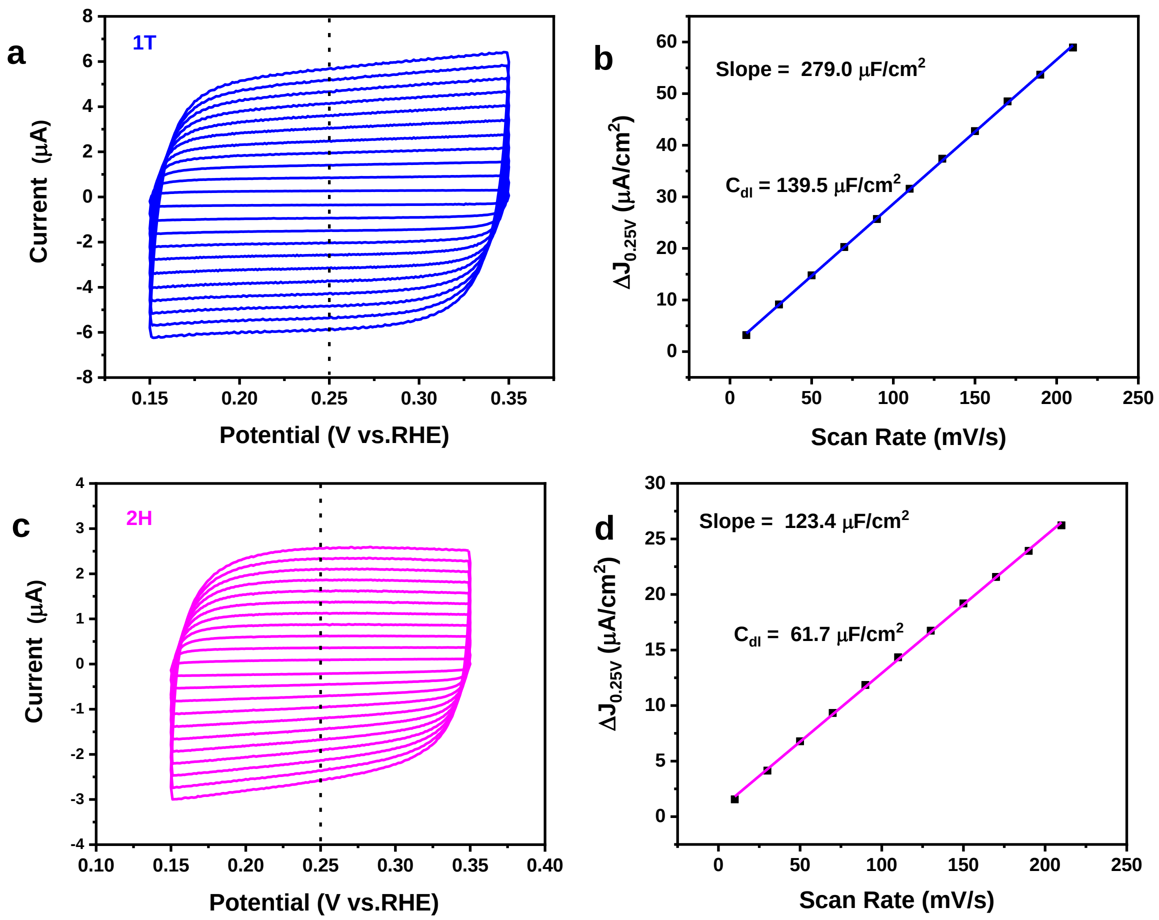


**Supplementary Fig. 47** Voltammograms of (a) V SACs@1T-$WS_2$ and (c) 2H-$WS_2$ electrocatalysts at various scan rates (10 ~ 210 mV/s); Electrochemically active surface area (b) V SACs@1T-$WS_2$ and (d) 2H-$WS_2$ estimated from the voltammograms at various scan rates (10 ~ 210 mV/s).

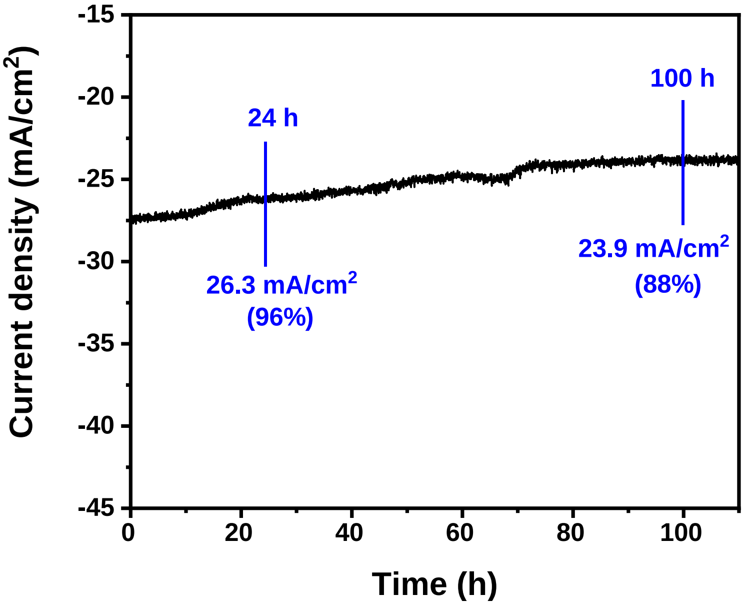


**Supplementary Fig. 48** Chronoamperometric curve of V SACs 1T-$WS_2$ catalyst at an overpotential of 400 mV in 0.5 M $H_2SO_4$ electrolyte.

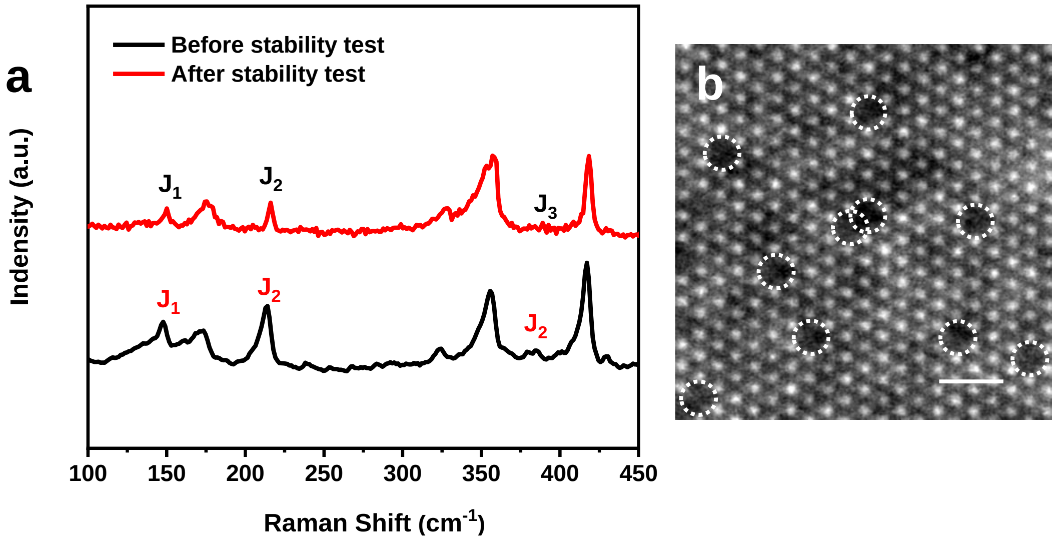


**Supplementary Fig. 49** Raman spectra (a) and STEM image (b) of V SACs 1T-$WS_2$ catalyst after stability test. The V SACs 1T-$WS_2$ catalyst after stability test was transferred on the sapphire substrate and TEM grid for the Raman spectrum and STEM measurements, respectively. The white dashed circles represent the V SACs. Scale bar: 1 nm.

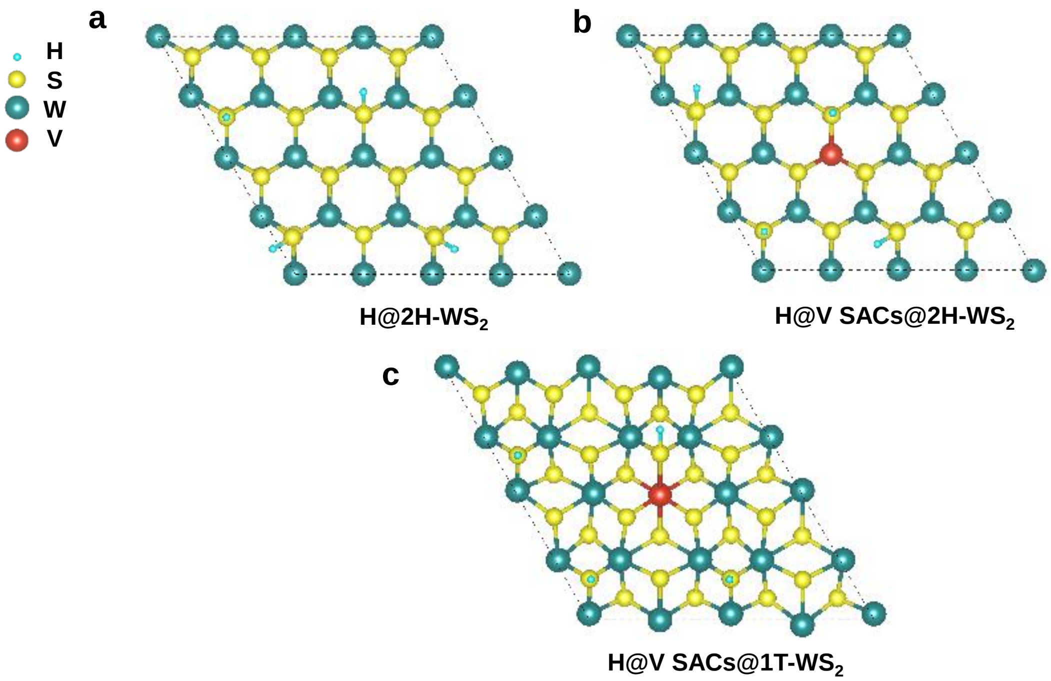


**Supplementary Fig. 50** Model structures of the 25% H-covered surface sites of 2H-$WS_2$ (a), V SACs@2H-$WS_2$ (b) and V SACs@1T-$WS_2$ (c). Since it has been demonstrated that 25% hydrogen coverage is the optimal coverage in terms of H binding for 1T-$MoS_2$,[44] here both non- and 25% H coverage was considered for the basal plane sites of the three studied phases.

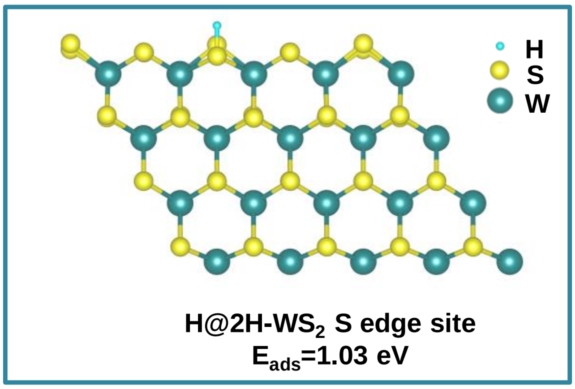


**Supplementary Fig. 51** Model structure of H adsorption on S edge sites of 2H-$WS_2$ with corresponding adsorption energy.

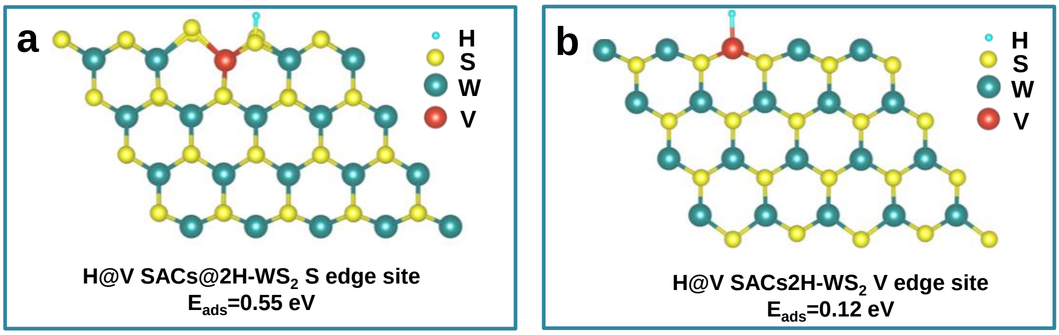


**Supplementary Fig. 52** Model structure of H adsorption on (a) S and (b) V edge sites of V SACs@2H-$WS_2$ with corresponding adsorption energies.

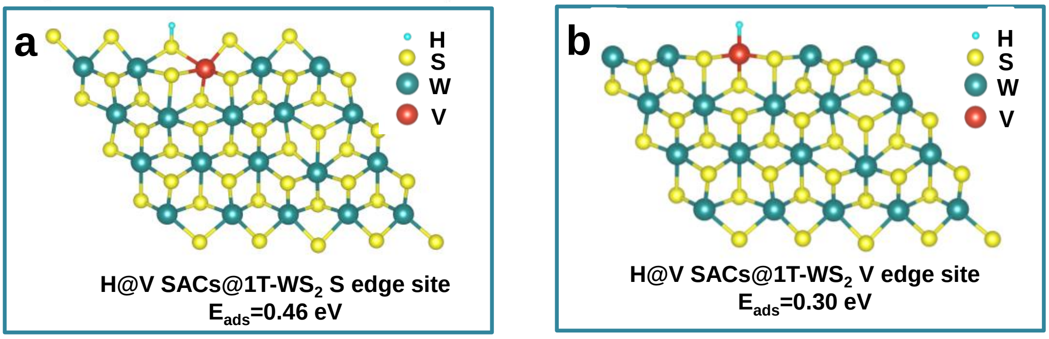


**Supplementary Fig. 53** Model structure of H adsorption on (a) S and (b) V edge sites of V SACs@1T-$WS_2$ with corresponding adsorption energies.

**Supplementary Table 1.** Comparison of the concentration of metallic $MX_2$ ($MoS_2$ or $WS_2$) via different strategies.

| Catalysts | Synthesis strategy | 1T phase content | 1T' phase content | References |
|---|---|---|---|---|
| **V SACs@1T-$WS_2$** | **One-step CVD** | **91%** | **-** | **In this work** |
| Strained 1T' $WS_2$ | Liquid phase exfoliated | - | 80% | Nat. Mater. 2013, 12 (9), 850-855. |
| 1T'- $MoS_2$ | Gas sulfidation plus $I_2$ treatment | - | 90% | Nat. Chem. 2018. 10, 638–643 |
| 1T'-$MoS_2$ | One-step CVD | - | 90% | Nat. Mater. 2018. 17. 1108. |
| 1T'-$MoS_2$ | Liquid phase exfoliated | - | 97% | Adv. Mater. 2019, 1900568 |
| 1T-$MoS_2$ | CVD plus post-exfoliated treatment | 80% | - | J. Am. Chem. Soc. 2013, 135, 10274 |
| 1T'-$MoS_2$ | Exfoliated synthesis plus $I_2$ treatment | - | - | Nano Lett. 2013, 13, 12, 6222-6227 |
| P-1T-$MoS_2$ | Liquid phase exfoliated | 82% | - | J. Am. Chem. Soc. 2016, 138, 7965−7972 |
| (N, $PO_4^{3-}$)-1T-$MoS_2$ | Hydrothermal | 41% | - | Angew. Chem. Int. Ed. 2019, 58,2 – 10 |
| 1T-$MoS_2$/$NiS_2$ | Hydrothermal | 83% | - | Angew. Chem. Int. Ed. 2019, 58,1 – 5 |
| 1T-$MoS_2$ | Liquid phase exfoliation | 70% | - | Nat. Nanotech. 2015, 10, 313. |

**Supplementary Table 2.** W4f$_{7/2}$ ratios of 1T and 2H under different annealing temperature. Relative percentages of 1T and 2H species obtained by integration of the W4f$_{7/2}$ peak for the four samples described in the main text Figure 3c and Supplementary Fig. S22

| $WS_2$ samples | 1T (W4f) | 2H (W4f) |
|---|---|---|
| Not annealed (25 °C) | 91% | 9% |
| 200 °C | 90% | 10% |
| 300 °C | 66% | 34% |
| 400 °C | 49% | 51% |

**Supplementary Table 3.** Computed lattice parameters of 2H-$WS_2$, V SACs@2H-$WS_2$ and V SACs@1T-$WS_2$.

| Samples | a | b | α | β | γ |
|---|---|---|---|---|---|
| 2H-$WS_2$ | 12.62 | 12.62 | 90 | 90 | 120 |
| V SACs@2H-$WS_2$ | 12.65 | 12.65 | 90 | 90 | 120 |
| V SACs@1T-$WS_2$ | 12.97 | 12.97 | 89.78 | 90.22 | 120.97 |

*To simplify the model, we apply V%=6.25% (W:V=15:1)

**Supplementary Table 4.** Statistics of $WS_2$ distribution with different lateral sizes in Supplementary Fig. 44d and the corresponding areas.

| Diameter Distribution (μm) | Mean diameter (μm) | Amount | Freq. | Area （μm$^2$） |
|---|---|---|---|---|
| 11-13.4 | 12.2 | 1 | 1.1% | 116.8 |
| 13.4-15.8 | 14.6 | 8 | 8.7% | 1338.6 |
| 15.8-18.2 | 17 | 10 | 10.9% | 2268.7 |
| 18.2-20.6 | 19.4 | 16 | 17.4% | 4727.1 |
| 20.6-23 | 21.8 | 22 | 23.9% | 8207.4 |
| 23-25.4 | 24.2 | 15 | 16.3% | 6895.9 |
| 25.4-27.8 | 26.6 | 9 | 9.8% | 4998.9 |
| 27.8-30.2 | 29 | 5 | 5.4% | 3300.9 |
| 30.2-32.6 | 31.4 | 3 | 3.3% | 2321.9 |
| 32.6-35 | 33.8 | 3 | 3.3% | 2690.4 |
| Total Area of $WS_2$ monolayers in Supplementary Fig. 44d | | | | 36866.6 |

*To calculate the area of $WS_2$ monolayers, we assume each $WS_2$ monolayer was circular and the mean diameter value represents the diameter (d) of circular. The area of each $WS_2$ monolayer was calculated according to the equation as follows:

$$Area\ of\ WS_2 monolayer = \pi r^2 = \frac{1}{4}\pi d^2$$

**Supplementary Table 5.** Statistics of $WS_2$ distribution with different lateral sizes in Supplementary Fig. 44e and the corresponding areas.

| Diameter Distribution (μm) | Mean diameter (μm) | Amount | Freq. | Area （μm²） |
|---|---|---|---|---|
| 10-13.1 | 11.55 | 1 | 1.3% | 104.7 |
| 13.1-16.2 | 14.65 | 4 | 5.3% | 673.9 |
| 16.2-19.3 | 17.75 | 8 | 10.5% | 1978.6 |
| 19.3-22.4 | 20.85 | 15 | 19.7% | 5118.9 |
| 22.4-25.5 | 23.95 | 15 | 19.7% | 6754.2 |
| 25.5-28.6 | 27.05 | 17 | 22.4% | 9764.6 |
| 28.6-31.7 | 30.15 | 8 | 10.5% | 5708.7 |
| 31.7-34.8 | 33.25 | 4 | 5.3% | 3471.5 |
| 34.8-37.9 | 36.35 | 3 | 3.9% | 3111.7 |
| 37.9-41 | 39.45 | 1 | 1.3% | 1221.7 |
| Total Area of $WS_2$ monolayers in Supplementary Fig. 44e | | | | 37908.5 |

**Supplementary Table 6.** Statistics of $WS_2$ distribution with different lateral sizes in Supplementary Fig. 44f and the corresponding areas

| Diameter Distribution (μm) | Mean diameter (μm) | Amount | Freq. | Area （μm²） |
|---|---|---|---|---|
| 13-15.3 | 14.15 | 1 | 1.2% | 157.2 |
| 15.3-17.6 | 16.45 | 6 | 7.4% | 1274.5 |
| 17.6-19.9 | 18.75 | 6 | 7.4% | 1655.9 |
| 19.9-22.2 | 21.05 | 14 | 17.3% | 4869.7 |
| 22.2-24.5 | 23.35 | 11 | 13.6% | 4708.0 |
| 24.5-26.8 | 25.65 | 18 | 22.2% | 9296.4 |
| 26.8-29.1 | 27.95 | 17 | 21.0% | 10425.1 |
| 29.1-31.4 | 30.25 | 3 | 3.7% | 2155.0 |
| 31.4-33.7 | 32.55 | 3 | 3.7% | 2495.1 |
| 33.7-36 | 34.85 | 2 | 2.5% | 1906.8 |
| Total Area of $WS_2$ monolayers in Supplementary Fig. 44f | | | | 38943.7 |

**Supplementary Table 7.** TOF values comparisons of representative electrocatalysts at different overpotentials in 0.5 M $H_2SO_4$.

| Electrocatalysts | Overpotential (mV) | TOF value ($s^{-1}$) | References |
|---|---|---|---|
| **V SACs@1T-$WS_2$** | **100** | **3.01** | **In this work** |
| **V SACs@1T-$WS_2$** | **150** | **12.78** | **In this work** |
| **V SACs@1T-$WS_2$** | **200** | **24.15** | **In this work** |
| SA Co-D-1T $MoS_2$ | 200 | ~19.0 | Nat. Commun. 2019, 10, 5231 |
| SA Co-HOPNC | 200 | 3.8 | Proc. Natl. Acad. Sci. USA 2018, 115, 12692. |
| SA Co-$P_1N_3$ | 100 | 1.6 | J. Am. Chem. Soc. 2020, 142, 8431−8439 |
| SA Co-$P_1N_3$ | 150 | 6.34 | J. Am. Chem. Soc. 2020, 142, 8431−8439 |
| Mo SAC | 150 | 1.46 | Angew. Chem. Int. Ed. 2017, 56, 16086. |
| P-1T-$MoS_2$ | 153 | 0.5 | J. Am. Chem. Soc. 2016, 138, 7965 |
| MoP | 150 | 0.19 | Angew. Chem. Int. Ed., 2014, 53, 14433-14437. |

* TOF values were calculated using the mass loading of 6.5 μg/$cm^2$.

**Supplementary Table 8.** Computed free energies of H adsorption ($|\Delta G_H|$) on surface, S and W/V edge sites for various species.

| $\|\Delta G_H\|$ (eV) | Surface site (basal plane) | S Edge site | W Edge site | V Edge site |
|---|---|---|---|---|
| 2H-$WS_2$ | 1.80 | 0.78 | 0.86 | - |
| V SACs@2H-$WS_2$ | 1.56 | 0.30 | 0.54 | 0.13 |
| V SACs@1T-$WS_2$ | 0.40 | 0.21 | 1.01 | 0.05 |

## References


1. Duerloo, K.-A. N., Li, Y. & Reed, E. J. Structural phase transitions in two-dimensional Mo-and W-dichalcogenide monolayers. *Nat. Commun.* **5**, 4214 (2014).
2. Gao, D. *et al.* Ferromagnetism in ultrathin $VS_2$ nanosheets. *J. Mater. Chem. C* **1**, 5909-5916 (2013).
3. Green, C. L. & Kucernak, A. Determination of the Platinum and Ruthenium Surface Areas in Platinum-Ruthenium Alloy Electrocatalysts by Underpotential Deposition of Copper. I. Unsupported Catalysts. *J. Phys. Chem. B* **106**, 1036-1047 (2002).
4. Wang, Y., Sofer, Z., Luxa, J. & Pumera, M. Lithium Exfoliated Vanadium Dichalcogenides ($VS_2$, $VSe_2$, $VTe_2$) Exhibit Dramatically Different Properties from Their Bulk Counterparts. *Adv. Mater. Interfaces* **3**, 1600433 (2016).
5. Zhou, J. *et al.* Hierarchical $VS_2$ Nanosheet Assemblies: A Universal Host Material for the Reversible Storage of Alkali Metal Ions. *Adv. Mater.* **29**, 1702061 (2017).
6. Yu, S. H. *et al.* In Situ Hybridizing $MoS_2$ Microflowers on $VS_2$ Microflakes in a One-Pot CVD Process for Electrolytic Hydrogen Evolution Reaction. *ACS Appl. Energy Mater.* **2**, 5799-5808 (2019).
7. Silversmit, G. *et al.* A comparative XPS and UPS study of VOx layers on mineral $TiO_2$ (001)-anatase supports. *Surf. Interface Anal.* **38**, 1257-1265 (2006).
8. Beams, R. *et al.* Characterization of Few-Layer 1T' $MoTe_2$ by Polarization-Resolved Second Harmonic Generation and Raman Scattering. *ACS Nano* **10**, 9626-9636 (2016).
9. Liu, L. *et al.* Phase-selective synthesis of 1T' $MoS_2$ monolayers and heterophase bilayers. *Nat. Mater.* **17**, 1108-1114 (2018).
10. Wang, Y. *et al.* Structural phase transition in monolayer $MoTe_2$ driven by electrostatic doping. *Nature* **550**, 487 (2017).
11. Munisso, M. C., Zhu, W. & Pezzotti, G. Raman tensor analysis of sapphire single crystal and its application to define crystallographic orientation in polycrystalline alumina. *Phys. Status Solidi B* **246**, 1893-1900 (2009).
12. Kafizas, A., Hyett, G. & Parkin, I. P. Combinatorial atmospheric pressure chemical vapour deposition (cAPCVD) of a mixed vanadium oxide and vanadium oxynitride thin film. *J. Mater. Chem.* **19**, 1399-1408 (2009).
13. Kuroda, N. & Fan, H. Y. Raman scattering and phase transitions of $V_2O_3$. *Phys. Rev. B* **16**, 5003-5008 (1977).
14. Xu, H. F., Liu, Y., Wei, N. & Jin, S. W. From $VO_2$(B) to $VO_2$(A) nanorods: Hydrothermal synthesis, evolution and optical properties in $V_2O_5H_2C_2O_4H_2O$ system. *Optik* **125**, 6078-6081 (2014).
15. Voiry, D. *et al.* Covalent functionalization of monolayered transition metal dichalcogenides by phase engineering. *Nat. Chem.* **7**, 45-49 (2015).
16. Allimi, B. S. *et al.* Growth of $V_2O_3$ thin films on a-plane (110) and c-plane (001) sapphire via pulsed-laser deposition. *J. Mater. Res.* **22**, 2825-2831 (2007).
17. Sakai, J., Limelette, P. & Funakubo, H. Transport properties and c/a ratio of

$V_2O_3$ thin films grown on C- and R- plane sapphire substrates by pulsed laser deposition. *Appl. Phys. Lett.* **107**, 241901 (2015).
18 Sun, G., Cao, X., Long, S., Li, R. & Jin, P. Optical and electrical performance of thermochromic $V_2O_3$ thin film fabricated by magnetron sputtering. *Appl. Phys. Lett.* **111**, 053901 (2017).
19 Allimi, B. S. *et al.* Resistivity of $V_2O_3$ thin films deposited on a-plane (110) and c-plane (001) sapphire by pulsed laser deposition. *Appl. Phys. Lett.* **92**, 202105 (2008).
20 Nag, N. K. & Massoth, F. E. ESCA and gravimetric reduction studies on $V/Al_2O_3$ and $V/SiO_2$ catalysts. *J. Catal.* **124**, 127-132 (1990).
21 Liang, H. *et al.* Solution Growth of Vertical $VS_2$ Nanoplate Arrays for Electrocatalytic Hydrogen Evolution. *Chem. Mater.* **28**, 5587-5591 (2016).
22 Yuan, J. *et al.* Facile synthesis of single crystal vanadium disulfide nanosheets by chemical vapor deposition for efficient hydrogen evolution reaction. *Adv. Mater.* **27**, 5605-5609 (2015).
23 Colton, R. J., Guzman, A. M. & Rabalais, J. W. Electrochromism in some thin-film transition-metal oxides characterized by x-ray electron spectroscopy. *J. Appl. Phys.* **49**, 409-416 (1978).
24 Ahmed, S. *et al.* Inducing High Coercivity in $MoS_2$ Nanosheets by Transition Element Doping. *Chem. Mater.* **29**, 9066-9074 (2017).
25 Poirier, L. *et al.* Thermal behavior of vanadocene. *J. Anal. Appl. Pyrolysis* **36**, 121-136 (1996).
26 Chow, P. K. *et al.* Wetting of Mono and Few-Layered $WS_2$ and $MoS_2$ Films Supported on $Si/SiO_2$ Substrates. *ACS Nano* **9**, 3023-3031 (2015).
27 van der Zande, A. M. *et al.* Grains and grain boundaries in highly crystalline monolayer molybdenum disulphide. *Nat. Mater.* **12**, 554-561 (2013).
28 Perea-López, N. *et al.* Photosensor Device Based on Few-Layered $WS_2$ Films. *Adv. Funct. Mater.* **23**, 5511-5517, doi:10.1002/adfm.201300760 (2013).
29 Zhang, Q. *et al.* Reliable Synthesis of Large-Area Monolayer $WS_2$ Single Crystals, Films, and Heterostructures with Extraordinary Photoluminescence Induced by Water Intercalation. *Adv. Optical Mater.* **6**, 1701347 (2018).
30 Ji, Q. *et al.* Unravelling Orientation Distribution and Merging Behavior of Monolayer $MoS_2$ Domains on Sapphire. *Nano Lett.* **15**, 198-205 (2015).
31 Yu, H. *et al.* Wafer-Scale Growth and Transfer of Highly-Oriented Monolayer $MoS_2$ Continuous Films. *ACS Nano* **11**, 12001-12007 (2017).
32 Voiry, D. *et al.* Enhanced catalytic activity in strained chemically exfoliated $WS_2$ nanosheets for hydrogen evolution. *Nat. Mater.* **12**, 850-855 (2013).
33 Yu, Y. *et al.* High phase-purity 1T'-$MoS_2$- and 1T'-$MoSe_2$-layered crystals. *Nat. Chem.* **10**, 638-643 (2018).
34 Sokolikova, M. S., Sherrell, P. C., Palczynski, P., Bemmer, V. L. & Mattevi, C. Direct solution-phase synthesis of 1T' $WSe_2$ nanosheets. *Nat. Commun.* **10**, 712 (2019).
35 Zhu, J. *et al.* Boundary activated hydrogen evolution reaction on monolayer $MoS_2$. *Nat. Commun.* **10**, 1348 (2019).

36 Wang, X. *et al.* Single-Atom Vacancy Defect to Trigger High-Efficiency Hydrogen Evolution of $MoS_2$. *J. Am. Chem. Soc.*, doi:10.1021/jacs.9b12113 (2020).

37 Lukowski, M. A. *et al.* Enhanced Hydrogen Evolution Catalysis from Chemically Exfoliated Metallic $MoS_2$ Nanosheets. *J. Am. Chem. Soc.* **135**, 10274-10277 (2013).

38 Yin, Y. *et al.* Contributions of Phase, Sulfur Vacancies, and Edges to the Hydrogen Evolution Reaction Catalytic Activity of Porous Molybdenum Disulfide Nanosheets. *J. Am. Chem. Soc.* **138**, 7965-7972 (2016).

39 Deng, S. *et al.* Synergistic Doping and Intercalation: Realizing Deep Phase Modulation on $MoS_2$ Arrays for High-Efficiency Hydrogen Evolution Reaction. *Angew. Chem. Int. Ed.* **58**, 16289-16296 (2019).

40 Qi, K. *et al.* Single-atom cobalt array bound to distorted 1T $MoS_2$ with ensemble effect for hydrogen evolution catalysis. *Nat. Commun.* **10**, 5231 (2019).

41 Zhang, H., Yu, L., Chen, T., Zhou, W. & Lou, X. W. Surface Modulation of Hierarchical $MoS_2$ Nanosheets by Ni Single Atoms for Enhanced Electrocatalytic Hydrogen Evolution. *Adv. Funct. Mater.* **28**, 1807086 (2018).

42 Meng, X. *et al.* Distance synergy of $MoS_2$-confined Rh atoms for highly efficient hydrogen evolution. *Angew. Chem. Int. Ed.*, doi:10.1002/anie.202003484 (2020).

43 Kim, H.-U. *et al.* Wafer-Scale and Low-Temperature Growth of 1T-$WS_2$ Film for Efficient and Stable Hydrogen Evolution Reaction. *Small* **16**, 1905000 (2020).

44 Tang, Q. & Jiang, D.-e. Mechanism of Hydrogen Evolution Reaction on 1T-$MoS_2$ from First Principles. *ACS Catal.* **6**, 4953-4961 (2016).
